# Bath: a Bayesian approach to analyze epigenetic transitions reveals a dual role of H3K27me3 in chondrogenesis

**DOI:** 10.1186/s13072-025-00594-6

**Published:** 2025-06-27

**Authors:** Christoph Neu, Manuela Wuelling, Christoph Waterkamp, Daniel Hoffmann, Andrea Vortkamp

**Affiliations:** 1 Developmental Biology, University Duisburg-Essen, Universitätsstr. 2, Essen, 45141 NRW Germany; 2 Bioinformatics and Computational Biophysics, University Duisburg-Essen, Universitätsstr. 2, Essen, 45141 NRW Germany

**Keywords:** Epigenetic regulation, Histone modification, Chondrocyte differentiation, Chromatin states

## Abstract

**Background:**

Histone modifications are key epigenetic regulators of cell differentiation and have been intensively studied in many cell types and tissues. Nevertheless, we still lack a thorough understanding of how combinations of histone marks at the same genomic location, so-called chromatin states, are linked to gene expression, and how these states change in the process of differentiation. To receive insight into the epigenetic changes accompanying the differentiation along the chondrogenic lineage we analyzed two publicly available datasets representing (1) the early differentiation stages from embryonic stem cells into chondrogenic cells and (2) the direct differentiation of mature chondrocyte subtypes.

**Results:**

We used ChromHMM to define chromatin states of 6 activating and repressive histone marks for each dataset and tracked the transitions between states that are associated with the progression of differentiation. As differentiation-associated state transitions are likely limited to a reduced set of genes, one challenge of such global analyses is the identification of these rare transitions within the large-scale data. To overcome this problem, we have developed a relativistic approach that quantitatively relates transitions of chromatin states on defined groups of tissue-specific genes to the background. In the early lineage, we found an increased transition rate into activating chromatin states on mesenchymal and chondrogenic genes while mature chondrocytes are mainly enriched in transition between activating states. Interestingly, we also detected a complex extension of the classical bivalent state (H3K4me3/H3K27me3) consisting of several activating promoter marks besides the repressive mark H3K27me3. Within the early lineage, mesenchymal and chondrogenic genes undergo transitions from this state into active promoter states, indicating that the initiation of gene expression utilizes this complex combination of activating and repressive marks. In contrast, at mature differentiation stages the inverse transition, the gain of H3K27me3 on active promoters, seems to be a critical parameter linked to the initiation of gene repression in the course of differentiation.

**Conclusions:**

Our results emphasize the importance of a relative analysis of complex epigenetic data to identify chromatin state transitions associated with cell lineage progression. They further underline the importance of serial analysis of such transitions to uncover the diverse regulatory potential of distinct histone modifications like H3K27me3.

**Supplementary Information:**

The online version contains supplementary material available at 10.1186/s13072-025-00594-6.

## Introduction

Epigenetic modifications of chromatin-associated proteins, particularly histones, are key to cell differentiation and identity. Over the last decade, genome-wide unbiased analyses like chromatin immunoprecipitation followed by high throughput sequencing (ChIP-Seq) have been established to investigate the genomic localization of histone modifications in many different cell types and tissues. Furthermore, computational tools like HMMSeg, ChromHMM, and Segway allow the integration of multiple datasets to identify combinations of epigenetic marks, so-called chromatin states [17, 21, 51]. Systematic large-scale studies conducted by consortia like *ENCODE* and *Roadmap* associated many of these states with functional genomic regions [33, 59]. For example, these studies found an accumulation of H3K4me3 and H3K9ac on active promoter regions, H3K27ac on active promoters and enhancers, H3K36me3 on transcribed gene bodies, and H3K27me3 and H3K9me3 on repressed chromatin regions [25, 55, 64, 67, 75].

While such epigenetic patterns are robust and reappear in different tissues and species, it is less well understood if and how transitions between chromatin states regulate distinct steps of differentiation within a cell lineage [12]. To receive insight into the dynamics of chromatin states, we have recently started to study how the epigenetic profile of chondrocytes changes during endochondral ossification. In this process, mesenchymal stem cells (MSC) differentiate into proliferating (PC) and, subsequently, hypertrophic chondrocytes (HC), which are later replaced by bone. This process takes place during embryonic development and in the repair of postnatal, unstabilized fractures, and it can also be replicated in cell culture [41, 60]. While MSC can be of different origins, the sequence of chondrogenic gene expression is highly conserved. A critical differentiation step is the switch from PC to HC, which is tightly controlled by many genetic and epigenetic factors [31, 58, 68, 70]. A comparison of the epigenetic signatures of murine PC and HC strongly suggested that the gain of repressive mark H3K27me3 on promoters that still carry several activating marks is a crucial event associated with the initiation of gene repression in HC [71]. To further test this hypothesis, we extended our analysis towards earlier differentiation stages by including MSC with chondrogenic potential as demonstrated by Wu et al. [69]. Additionally, we investigated how the chondrogenic lineage is established by examining the differentiation of embryonic stem cells (ESC) into MSC and MSC-derived chondrogenic cells.

The global analysis of changes in the epigenetic pattern associated with distinct steps of differentiation is complicated by the restricted number of genes expressed in the chondrogenic lineage and the fact that only a subset of these genes alters their expression at a given differentiation step. Consequently, in genome-wide analyses, these rare events are prone to be masked by changes in the overall epigenetic pattern, especially if data of different origins are included. To overcome this problem, we have focused our analysis on groups of unbiasedly chosen, tissue-specific genes. To facilitate the transition analysis, we have created BATH (Bayesian Analysis for Transitions of Histone States), a Bayesian-based computational tool designed for the quantitative analysis of chromatin state dynamics between different cell types. BATH determines the probability with which a gene of a given group shows a distinct transition between two states and relates this information to the probability of this transition occurring in the background.

The quantitative analysis of chondrocyte-specific chromatin state transitions strongly supports the hypothesis that the combination of the repressive mark H3K27me3 with the activating marks H3K4me3, H3K9ac, and H3K27ac is closely linked to alterations in gene expression during chondrocyte differentiation. Interestingly, we could identify the loss of H3K27me3 to be connected with the establishment of the early chondrogenic lineage, while in mature chondrocyte subtypes the gain of H3K27me3 is associated with gene repression, highlighting the dynamic role of H3K27me3 in the progression of cell differentiation.

## Methods

All scripts for automatic data retrieval, processing, and information on the used software are available at https://gitlab.com/kEks/bath. The analysis can be reproduced using a processing pipeline based on snakemake, conda, and containers [1, 42, 44].

### Preprocessing of ChIP-Seq data

For each analysis, ChIP-Seq data of 6 histone modifications (H3K4me3, H3K9ac, H3K27ac, H3K36me3, H3K9me3, and H3K27me3) were used.

For the early chondrogenic lineage (ECL), we analyzed 4 human cell types: H1 Cells E003 (ESC), H1 Derived **M**esenchymal **S**tem **C**ells E006 (eMSC), **B**one **M**arrow-Derived Cultured **M**esenchymal **S**tem **C**ells E026 (bmMSC) and **C**hondro**c**ytes from **B**one **M**arrow Derived Mesenchymal Stem Cell Cultured Cells E049 (bmCC). The peak-called files of each replicate and the consensus data, combining the replicates of a given mark and cell type, were downloaded from the Roadmap Epigenomics Consortium [33]. For the mature chondrogenic lineage (MCL) data, we analyzed Chip-Seq data of FACS-isolated embryonic PC and HC [71] and included MSC data of bone marrow-derived primary MSC with chondrogenic potential, isolated from femurs and tibias of 6-8 week old SMAA-mCherry mice [69], retrieved from the Sequence Read Archive (SRA, Supp.Table. A1). Murine and human data were processed separately with two snakemake (6.10.0, [44]) pipelines. For the MCL data, the fastq files were trimmed (trimmomatimc 0.39, [11]) aligned to mm39 (bwa 0.7.17, [35]), and filtered for quality, duplication, and somatic chromosomes (samtools 1.9, [36]). Gonosomes were excluded as the sex of the mice was unknown. Every step was quality controlled before (fastQC 0.11.9, [2]) and after (qualimap 2.2.2a, [45]) alignment. Peaks were called with hiddenDomains (3.1, [53]) with a bin size of 200 bp (H3K4me3, H3K9ac, and H3K27ac) or 800 bp (H3K27me3, H3K36me3, and H3K9me3). Neighboring peaks with a distance of less than three bins were merged with a sliding window approach. As a control, IgG sequencing data were used for PC and HC, while not immunoprecipitated samples (Input) were used for MSC. Peak-called ECL data were derived from the Roadmap Epigenomics Consortium and the gonosomes were removed. To estimate sample similarities, the Jaccard index was calculated for every pair of samples using bedtools (2.26.0, [28], see Eq. 1).

1 $${\text{Jaccard}}(BedFileA,BedFileB) = \frac{{\# basepairs(Intersection)}}{{\# basepairs(Union)}}{\text{ }}$$

To obtain the most robust combinations of epigenetic marks, the consensus peaks (intersection of the respective replicates per histone mark and cell type) were used to train a hidden Markov model (ChromHMM 1.20, [21]). We tested hidden Markov models with the number of states ranging from 5 to 30. The model with 15 states was selected for further work as it combined high log-likelihood with a clear biological interpretation of states. For each of the 15 states the mark emissions, the localization at distinct genomic regions (genome, gene, exon, CpG island, transcriptional start site (TSS), transcriptional end site (TES)), and the distribution around the anchor points TSS and TES provided the basis for the state annotation. For the human dataset, the default hg19 reference data of ChromHMM was used, while for the mouse data a custom reference dataset was created to allow mm39 annotation (https://hgdownload.soe.ucsc.edu/goldenPath/). To account for sample variation, the states learned on the consensus peaks were subsequently assigned to the individual replicates with the ChromHMM commands *BinarizeSignal* and *MakeSegmentation*. Overlap of a signal for a given sample to a set of genes was determined with bedtools for the peak-called data and the ChromHMM states.

A humanized murine epigenome was created by assigning the human ChromHMM model to the murine replicates (*MakeSegmentation*). For each replicate, we calculated the Jaccard index (see Eq. 1) of every combination of states between the humanized and murine model (e.g. each state of the murine replicate A with each state of the humanized replicate A).

To visualize chromatin state coverage, the MCL ChromHMM data were uploaded to the UCSC genome browser (http://genome.ucsc.edu [46]). Information on all genes can be accessed via: https://genome.ucsc.edu/s/manuela.wuelling%40uni%2Ddue.de/mm39_Murine_humanizedChromHMM.

### Gene sets

Gene sets of interest were generated by providing gene lists: GO term IDs for genes associated with mesenchymal (78+7) and chondrogenic gene (105+7) function [49] (Supp.Table. A2) were fetched by biomart (2.48.0, [19, 20]). To obtain tissue-specific sets, seven genes that belong to both, the mesenchymal and chondrogenic set, were removed. As controls, we used a list of 270 housekeeping genes, derived from de Jonge et al. [29], and sets of 40, 50, and 100 random genes sampled from the reference genome (Ensembl Archive Release 104). To check for biases due to gene size, the length distributions of the gene sets were compared and found to be similar to those of the background (data not shown). For a visualization of the whole process see Supp.Fig. B1A.

### Calculation of state transition enrichment

The processing of the ChromHMM state data and computation of transition enrichment and depletion was performed with a snakemake pipeline, which was parameterized with config files in YAML and csv format.

For each combination of samples (replicates and cell types), every state transition at a given location (bin) was determined and assigned to the genes covering this bin. The direction of the transition (cell type from which the transition originates to the cell type it settles in) was assigned based on the direction of differentiation. For the replicates of a given cell type, the order was randomly assigned.

Genes were categorized into the previously defined sets, and for every combination of state pairs, gene sets, and cell type pairs the number of genes with a given transition was determined. To evaluate if a transition for a given category of genes, was more or less frequent compared to the background (the set *a* of all genes), a Bayesian hierarchical beta-binomial model was fitted with Stan 2.21.2 [14] (Eq. 2-9).

2 $$\begin{aligned} s^{(g)}_{t, d, i, j}&\sim \text {BetaBin}\left( S^{(g)}, \alpha ^{(g)}_{t, d}, \beta ^{(g)}_{t, d} \right) \end{aligned}$$

3 $$\begin{aligned} a_{t, d, i, j}&\sim \text {BetaBin} \left( A , \alpha ^{(a)}_{t, d} , \beta ^{(a)}_{t, d} \right) \end{aligned}$$

4 $$\begin{aligned} \alpha ^{(g)}_{t, d}&\sim \text {Exponential}(\alpha _h) \end{aligned}$$

5 $$\begin{aligned} \beta ^{(g)}_{t, d}&\sim \text {Exponential}(\beta _h) \end{aligned}$$

6 $$\begin{aligned} \alpha ^{(a)}_{t, d}&\sim \text {Exponential}(\alpha _h) \end{aligned}$$

7 $$\begin{aligned} \beta ^{(a)}_{t, d}&\sim \text {Exponential}(\beta _h) \end{aligned}$$

8 $$\begin{aligned} \alpha _h&\sim \text {Exponential}(10) \end{aligned}$$

9 $$\begin{aligned} \beta _h&\sim \text {Exponential}(10) \end{aligned}$$

The number of genes ($s^{(g)}$ for gene set *g* and *a* for all genes) with a given transition (combination of states *t*) for a given differentiation step (combination of cell types *d*) was assumed to follow a beta-binomial distribution (Eqs. 2 and 3). The parameters of the distribution are the number of genes in the set (*S*) or the background (*A*), and two shape parameters, $\alpha$ and $\beta$. As prior probabilities of the shape parameters, we used exponential distributions (Eqs. 4-7). The corresponding rate hyperparameters ($\alpha _h$ and $\beta _h$) were modeled with exponential distributions with a rate of 10 (Eqs. 8 and 9) so that the preferentially small values produced broad, weakly informative prior distributions in Eqs. 4-7. The indices *i* and *j* indicate the unique identifiers for the replicates of a given combination. The convergence was verified by $\hat{R}$ values close to one and the quality of the fit was tested by posterior predictive checks (PPC) [61]. To summarize the PPC data the difference between the observed number of genes with a given transitions, against the models’ predictions was plotted.

To evaluate for every combination of state transition (*t*), cell differentiation (*d*), and set of genes (*g*) whether a deviation from the background exists, we calculated the common language effect size (CLES) [40]. The CLES expresses the expected outcome of a comparison between two representative genes, one from the set, and one from the background. Specifically, we compare the probability of the set gene relative to the background gene to have a given transition *t* in a given developmental step *d*. In other words, the CLES quantifies the enrichment or depletion of transitions *t* in the gene set in comparison to the background set as we take developmental step *d*.

We calculate the CLES in multiple stages, using the posterior distribution of the fitted Bayesian model. First, we estimate the probability that a single randomly selected gene from the background set *a* does *not* undergo a transition *t* in developmental step *d* (first factor in Eq. 10). Second, we estimate the probability that a single random gene in the selected set *g* *does* undergo that transition (second factor in Eq. 10). Multiplying both probabilities will provide *inc*. A high value of *inc* represents an enrichment of *t* in *g* relative to *a*. Equation 11 evaluates the corresponding tendency of a depletion (*dec*) of *t* in *g* relative to *a* (transition in the background, but not in the set). It is important to note that low values for the enrichment do not necessarily imply high values of depletion and low values of depletion do not necessarily imply high values of enrichment. To avoid reporting two values for each transition, we combine *inc* and *dec* in the CLES value (Eq. 12), providing a metric, which can be interpreted as a pairwise comparison of one representative gene of the set and one representative gene of the background. Values above 0.5 correspond to enrichment, i.e. a higher chance that the set gene shows a given transition in comparison with a representative background gene. Values below 0.5 represent depletion, i.e. a lower chance of the set gene showing the transition. For a visualization of the transitions and their processing to obtain the CLES value, see Supp.Fig. B1B.

10 $$\begin{aligned} inc^{(g)}_{t,d}&= \text {BetaBin}\left( 0 \,|\, 1, \alpha ^{(a)}_{t, d } , \beta ^{(a)}_{ t, d } \right) \cdot \text {BetaBin}\left( 1 \,|\, 1, \alpha ^{(g)}_{ t, d} , \beta ^{(g)}_{ t, d} \right) \end{aligned}$$

11 $$\begin{aligned} dec^{(g)}_{t, d}&= \text {BetaBin}\left( 1 \,|\, 1, \alpha ^{(a)}_{t, d} , \beta ^{(a)}_{ t, d}\right) \cdot \text {BetaBin}\left( 0 \,|\, 1, \alpha ^{(g)}_{ t, d} , \beta ^{(g)}_{ t, d} \right) \end{aligned}$$

12 $$\begin{aligned} \text {CLES}^{(g)}_{ t, d}&= \frac{inc^{(g)}_{ t, d} + (1-dec^{(g)}_{ t, d})}{2} \end{aligned}$$

Although this approach results in single-value effect sizes, the one-to-one comparison neglects variance, which is only meaningful if multiple genes are compared. In order to assess to what extent the variance affects the results, we calculated the CLES for a group size of 100 genes (the same magnitude as the used gene sets). For the vast majority of transitions, the variance does not influence the rank of the effect size (Supp.Fig. B2.). With both CLES values being highly correlated, the overall results remained the same. With this excellent agreement and the inherent arbitrariness in choosing a group size, this work focuses on the results of the one-to-one comparison.

## Results

To generate an epigenetic profile of the chondrogenic lineage we analyzed two ChIP-Seq datasets representing (1) the early differentiation stages from ESC to mesenchymal and chondrogenic cells, and (2) later differentiation stages from MSC to proliferating and hypertrophic chondrocytes. The dataset of early differentiation stages (ECL; early chondrogenic lineage) consists of 4 cell lines, which cover a broad differentiation spectrum but do not include specific chondrocyte cell types. Due to the lack of publicly available data of early mouse differentiation stages, in which the same set of histone marks has been investigated as in our previous study [71], we used human datasets of the Roadmap consortium: (1) embryonic stem cells (ESC), (2) MSC differentiated from ESC (eMSC), (3) bone marrow-derived MSC (bmMSC) and (4) chondrogenic cells differentiated from bmMSC (bmCC). To focus on direct differentiation steps within the chondrogenic lineage the second dataset (MCL; mature chondrogenic lineage) represents the differentiation of MSC derived from ribs of 6-week-old mice [69] into the defined chondrocyte subtypes of PC and HC, which were isolated by FACS sorting [71]. The chondrogenic potential of MSC has been confirmed by Wu et al. [69]. For all cell types, we analyzed 6 histone modifications covering the promoter region (H3K4me3, H3K9ac, H3K27ac), the transcribed gene body (H3K36me3), and repressed genomic regions (H3K27me3 and H3K9me3).

**Fig. 1:**
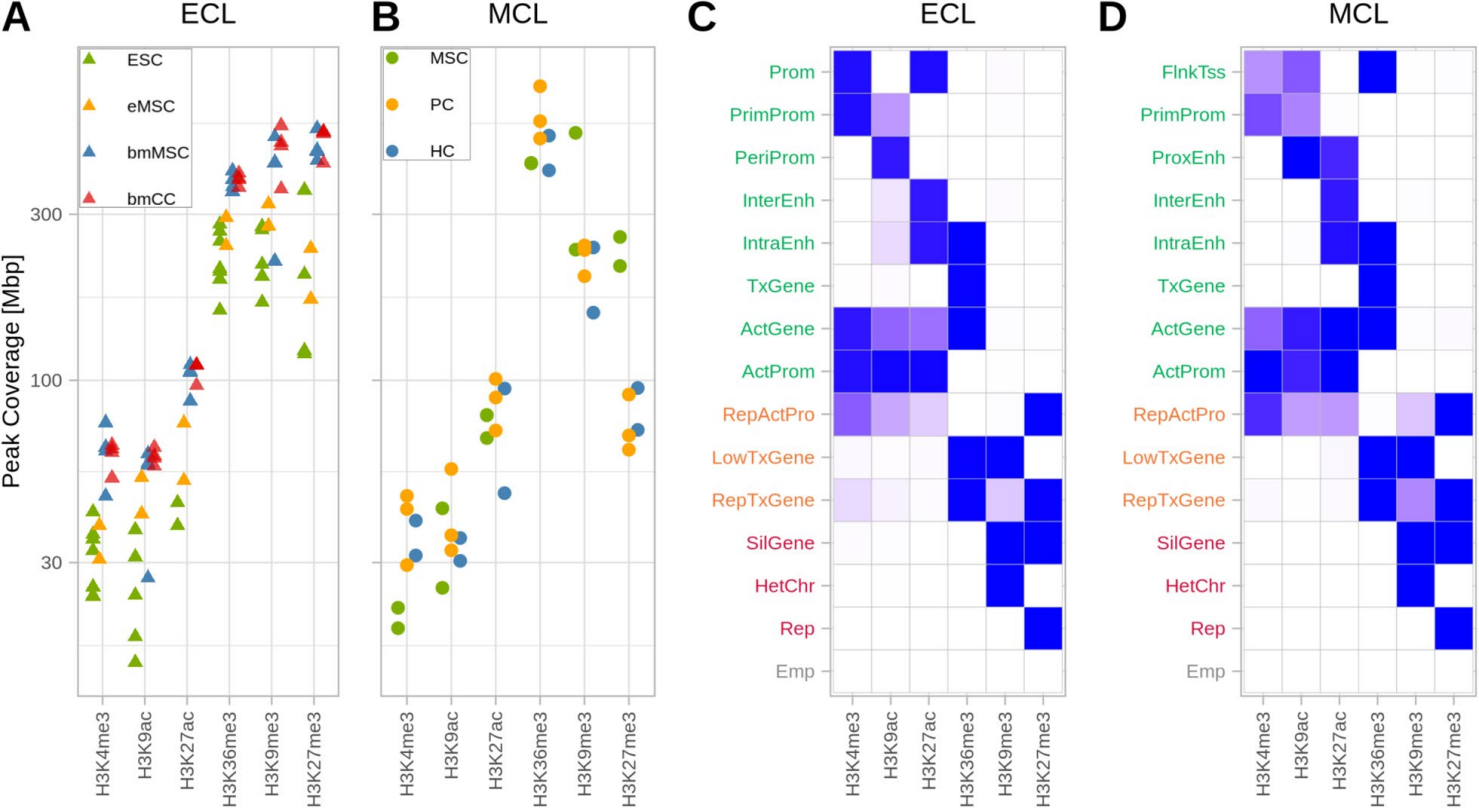
The coverage of histone marks and ChromHMM states are similar in both datasets. **A**-**B** Genomic coverage of the epigenetic marks for each cell type and replicate. Points are spread out horizontally by small displacements to reduce overplotting.** C**-**D** Emission probabilities of the 15-state ChromHMM model, based on the consensus data of the replicates. The colors of the row names categorize the states into four groups: activating (green), repressed-active (orange), repressive (red), and empty (grey)

As a first step, we evaluated the genomic coverage of the histone marks. Peak calling detected narrow peaks for the promoter marks, while H3K36me3, H3K27me3, and H3K9me3 demarcated broader genomic regions. Accumulating the peak length of each modification revealed a similar pattern in the respective cell types, with lower genomic coverage for promoter marks and considerably higher coverage for the broad marks. In the ECL data, the more differentiated, bone marrow-derived cell types clustered together, showing a generally higher level of modification than the ESC-derived samples (Fig. 1A). The cell types of the MCL showed a similar coverage for most histone modifications except for the coverage of the repressive mark H3K27me3, which was notably higher in MSC than in PC and HC (Fig. 1B).

To estimate the overall similarity of the data we calculated the Jaccard index of each pair of samples (Supp.Fig. B3A-B). In both datasets, the distribution of activating promoter marks was highly similar between each other, within and between the individual cell types. The marks characterized by broad peaks agreed well between cell types but differed from each other and from the promoter marks. Furthermore, for all marks of the ECL dataset, the ESC-derived cell types (ESC, eMSC) showed better agreement with each other than with the bone marrow-derived cells (bmMSC, bmCC), possibly reflecting their different biological origin (Supp.Fig. B3A). In the MCL dataset, the genomic localization of the repressive marks H3K27me3 and H3K9me3 was considerably different in MSC compared to PC and HC (Supp.Fig. B3B).

Understanding the role of epigenetic modifications at a distinct genomic position requires determining the combination of marks at the given location. For each dataset (ECL and MCL), we trained a hidden Markov model (“ChromHMM model”) on the consensus data of each histone mark per cell type (see flowchart in (Supp.Fig. B1A). Models with different numbers of chromatin states were evaluated based on their log-likelihood and the clarity of biological interpretation of the states (Supp.Fig. B4A,D). For each dataset, a 15-state model was chosen, and the respective states were annotated based on the mark emission, the localization at distinct genomic regions (genome, gene, exon, CpG island, TSS, (Supp.Fig. B4B,E)), the distribution around the anchor points (TSS and TES Supp.Fig. B4C,F), and the expected regulatory role of the accumulated marks [21, 24, 33, 62]. The models derived from each dataset were robust, with 13 out of 15 states occurring in both datasets (Fig. 1C-D). Besides non-decorated regions [empty states (Emp)] we defined six common states linked to active gene expression covering the promoter region [active gene (ActGene), active promoters (ActProm), and primed promoters (PrimProm)], the transcribed gene body [intragenic enhancers (IntraEnh), transcribed genes (TxGene)], and enhancer regions [intergenic enhancers (InterEnh)]. In addition, two activating states covering the promoter region differed between the datasets: The peripheral promoter (PeriProm) and promoter (Prom) states in the ECL data were substituted with the proximal enhancer (ProxEnh) and flanking TSS (FlnkTSS) states in the MCL data. In both datasets, three states were composed of only repressive marks [silenced genes (SilGene), heterochromatin (HetChr), and repressed regions (Rep)], and three states were characterized by a combination of activating and repressive marks [low transcribed genes (LowTxGene), repressed transcribed genes (RepTxGene) and repressed active promoter (RepActPro)].

Among these states, the RepActPro state is especially intriguing as it can be regarded as a variant of the classical bivalent state (H3K4me3/H3K27me3), which has been described to mark lineage-specific genes in ESC [4, 8]. Surprisingly, although ESC are part of the ECL data, the bivalent state did not emerge in the described 15-state ChromHMM model. It was, however, detected if only ESC were used to train a separate 15-state model (Supp.Fig. B5A-B). Nevertheless, in this model, the RepActPro state was also detected although with a lower prevalence than in the full ECL model.

While overall the distribution of states was quite similar between the ECL and MCL model, single states show a different emission of individual marks. For example, the low emission of H3K9me3 on the MCL RepActPro state was not included in the respective ECL state. As the ChromHMM models were derived from different species, this might indicate a species-specific effect. To test whether such a difference is critical for the identity of the state, we applied the ECL-derived, human ChromHMM model to the murine MCL replicates and compared it to the murine model by calculating the Jacquard index. The murine FlnkTSS and ProxEnh states weakly colocate with different promoter and enhancer states of the humanized model, likely reflecting the high dynamics in promoter-associated activating states. The 13 identically annotated states including the RepActPro state were almost exclusively assigned to the same genomic region in both models despite the slight difference in states emission (Supp.Fig. B6). Additionally, visualization of exemplary genes carrying the RepActPro state (Supp.Fig. B7) supported the similarity between the human and murine ChromHMM model.

As demonstrated above, the consensus data led to robust chromatin state models. At the same time, the use of consensus data does not account for the variance between replicates. However, this variance is important to assess whether a given difference in state coverage between two cell types simply reflects the background variation, or is linked to a specific role in one cell type. To include the variance between replicates the 15 ChromHMM states were mapped to the individual replicates of each mark and cell type (see flow chart in Supp.Fig. B1A). In the ECL data, the state coverage was clearly dependent on the cellular origin (ESC or bone-marrow-derived) (Supp.Fig. B5A), whereas the MCL data displayed an increased coverage of the H3K27me3-containing SilGene state in MSC (Supp.Fig. B5C). While the observed differences in state coverage were consistent with the pattern of the individual marks, it remains unclear if they reflect a cell type-specific, epigenetic decoration, related to lineage differentiation, or if they are due to technical variances of independently obtained data.

To distinguish between these alternatives, we analyzed the coverage of ChromHMM states on tissue-specific genes relative to the coverage on all protein-coding genes (Supp.Fig. B8). Based on GO terms [3, 15], we defined two groups of tissue-specific genes, a group of 78 mesenchymal and a group of 105 chondrogenic genes. Groups of 40, 50, or 100 random draws of background genes and a published group of 270 housekeeping genes [29] served as controls (Supp.Fig. B8).

**Fig. 2:**
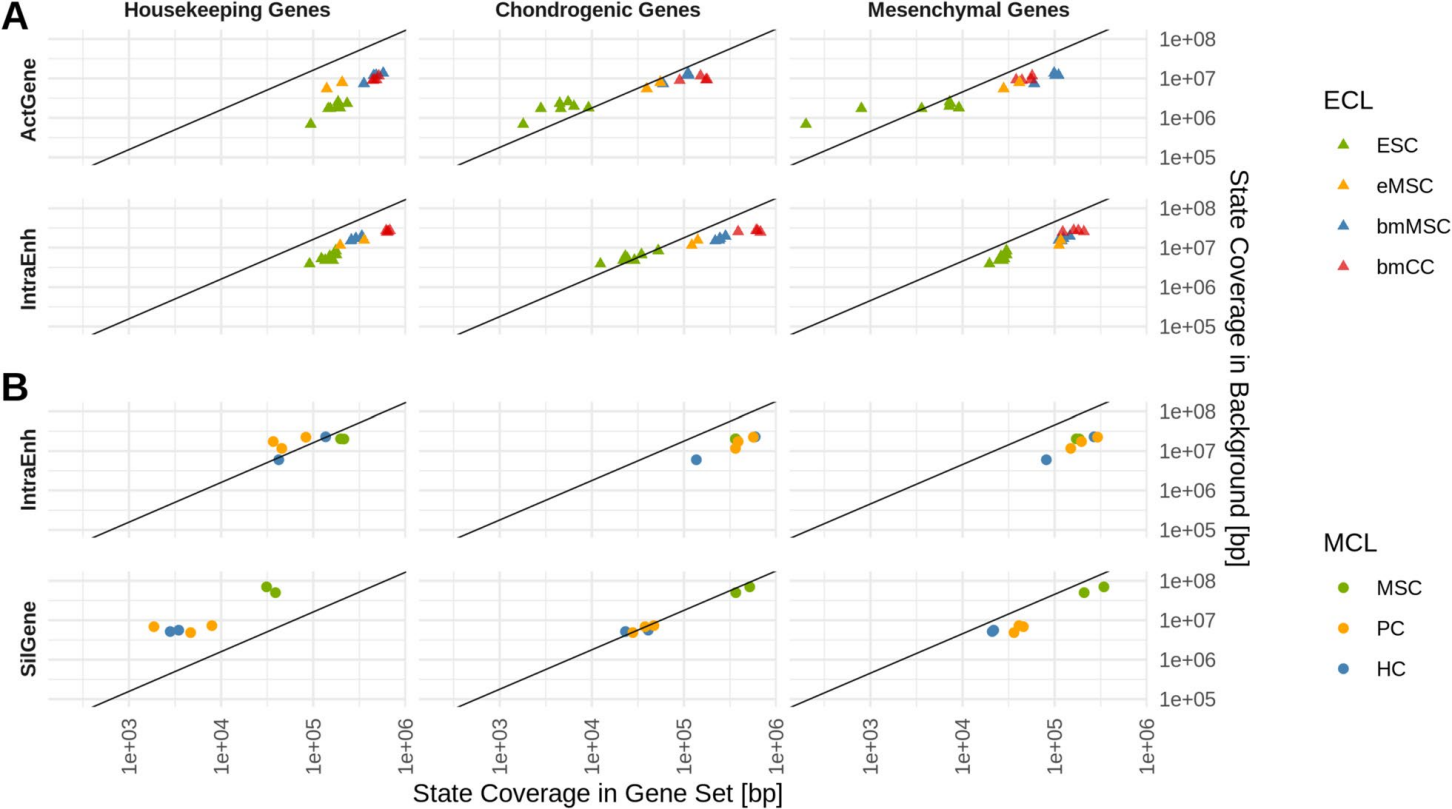
The relative coverage of ChromHMM states reveals systematic differences of epigenetic coverage between cell types. Coverage in base pairs of a given state (row) on a given gene set (column) was plotted against the coverage on all genes.** A** shows data of the ECL and (**B**) of the MCL. The black line (slope = [length of all background genes] / [length of all genes within a set], intercept= 0) delineates an equal proportional coverage of the background and the gene set. Deviation from the line indicates enrichment (below the line) or depletion (above the line) of the state in the respective gene set. See Supp. Fig. B8 for all combinations of gene sets and states

As expected, the state coverage on the randomly drawn genes did not deviate markedly from the background, especially for higher numbers of random genes. Similarly, the relative state coverage of the housekeeping and tissue-specific (mesenchymal, chondrogenic) genes did not differ considerably between the distinct cell types of each dataset (Fig. 2). Most notably the SilGene state, which has an increased prevalence in MSC compared to PC and HC, did not deviate from the background if relative state levels were considered. This strongly indicates that the increased coverage was not related to an epigenetic function, but rather represented a generally elevated decoration in MSC or a technical difference in data acquisition.

While the relative prevalence was similar between the cell types, closer inspection revealed deviations from the background for distinct states. This was most obvious for housekeeping genes, which were enriched in activating states (ActGene, ActProm) in all cell types, while repressive states like the SilGene state were underrepresented. Similar enrichment and depletion patterns were seen for mesenchymal and chondrogenic genes (Fig. 2). Interestingly, in the ECL data the relative prevalence of states of active transcription, like the ActGene, IntraEnh, and TxGene state, correlated with cellular maturation, especially for chondrogenic genes, switching from being underrepresented to an above-average decoration in further differentiated cells (Fig. 2, Supp.Fig. B8).

Taken together, our data emphasize the importance of a relative data analysis, in order to distinguish unspecific and potentially artificial effects from more nuanced, ‘true’ effects, associated with gene expression during cell differentiation.

**Fig. 3:**
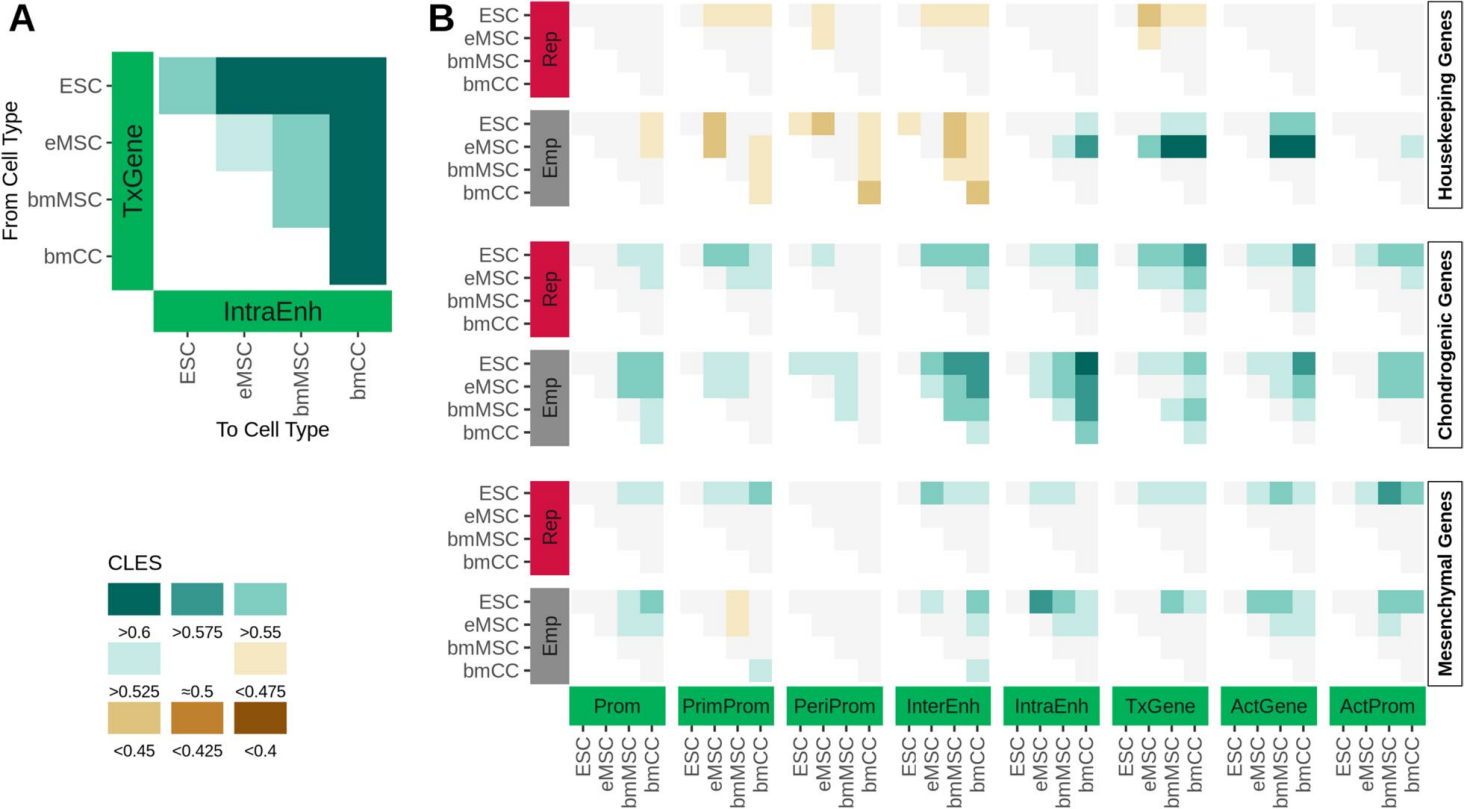
Systematic enrichment of activating marks during lineage establishment. Individual effect sizes for selected transitions of housekeeping, chondrogenic, and mesenchymal genes (CLES color key at the bottom left). Left and bottom axes represent the origin and target states, respectively, with state groups empty (gray), repressive (red), and activating (green). **A** Exemplary transition of the ECL data on housekeeping genes from the TxGene state to the IntraEnh state. This active-to-active state transition was enriched between all analyzed cell types. The enrichment was particularly strong from ESC into more mature cells and from every cell type into bmCC.** B** Selection of transition with drastic shifts of the epigenetic pattern, from empty or repressive states to activating states. See Supp.Fig. B12 for all combinations of states, cell types, and gene sets

Taking the relativistic approach one step further, we next aimed to understand how specific alterations in the epigenetic profile (transitions) were related to cell differentiation. For a quantitative answer that accounts for sample variation, we built a Bayesian model that calculates transition probabilities. In short, the model determines the probability with which a transition between a pair of states occurs between two cell types on a gene in the background or a gene of a given set. The direction of transition between replicates of the same cell type (e.g. MSC to MSC) was assigned arbitrarily and reflect the dynamics of decoration within the cell type. The direction between cell types was based on the direction of differentiation: from ESC to eMSC to bmMSC to bmCC for the ECL data, and from MSC to PC to HC for the MCL data. These transitions reflect changes that are linked to differentiation. The model passed all checks for convergence and validity (see Methods, Supp.Fig. B9, Supp.Fig. B10, and Supp.Fig. B11). The transition probabilities were expressed in terms of the common-language effect size value (CLES), a score between 0 and 1. Values below and above 0.5 indicate depletion and enrichment of transitions in the gene set in comparison to the background, respectively (Fig. 3A, Supp.Fig. B1B for graphical explanation).

**Fig. 4:**
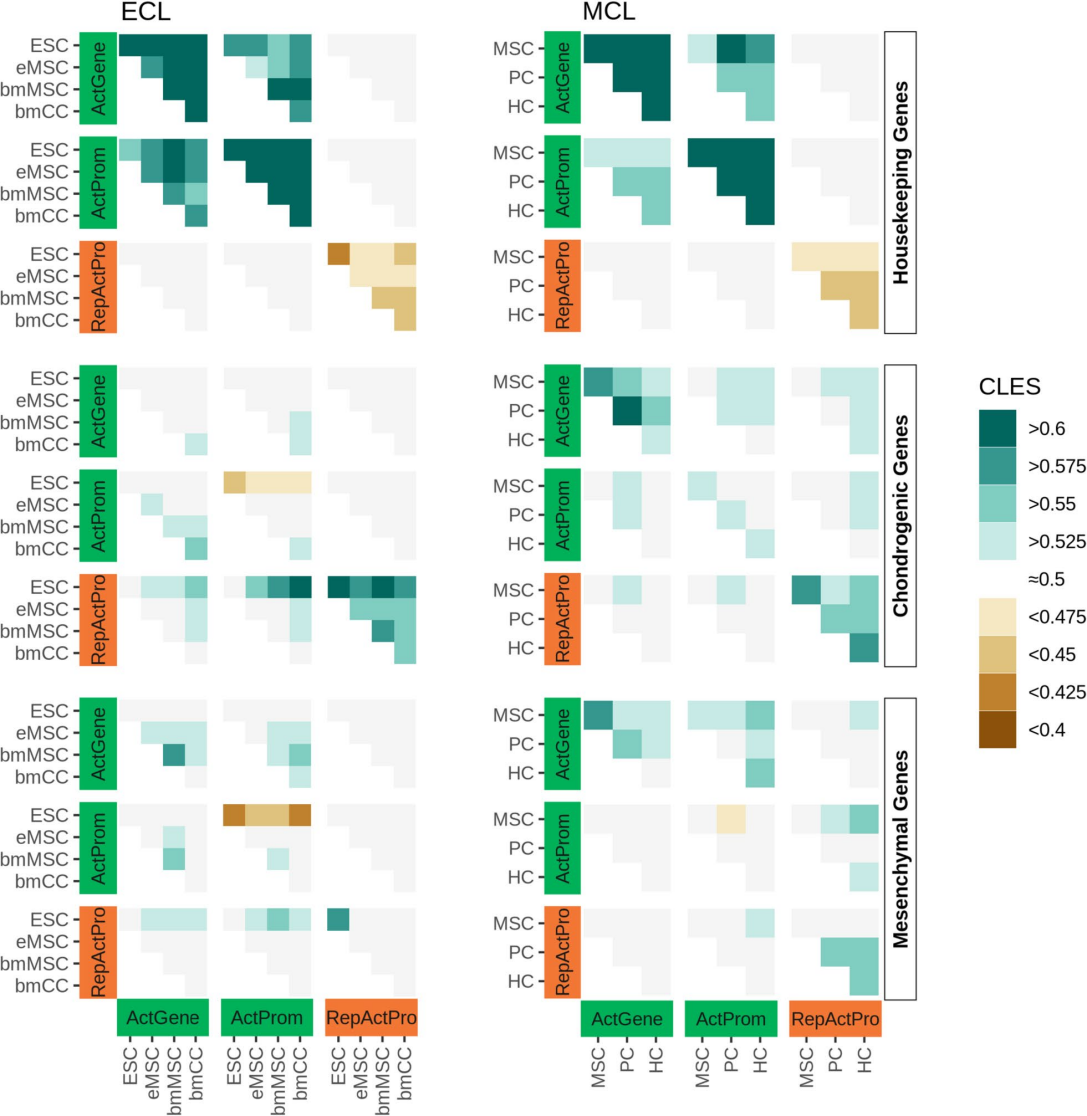
The formation and breakdown of the RepActPro state are directly linked to cellular differentiation. Housekeeping genes reveal a consistent pattern in ECL and MCL data, with clear enrichments between the activating promoter states (ActGene and ActProm; top left corners in the individual sections) and depletions for the persistence of the RepActPro state (bottom right corners in the individual sections). For chondrogenic and mesenchymal genes there is an enrichment in the formation of the activating states by losing H3K27me3 in the ECL (RepActPro to ActGene/ActProm; bottom rows in the individual sections). In contrast, in the MCL the formation of the RepActPro state by gaining H3K27me3 is more pronounced (ActGene/ActProm to RepActPro; right column in the individual sections). See Supp.Fig. B12 for all combinations of states, cell types, and gene sets

We first analyzed the control gene sets of 40, 50, and 100 randomly drawn genes. In these control sets, most transitions had effects with CLES values between 0.45 to 0.55. Especially with higher gene numbers barely any effects or discernible patterns were visible. In contrast, much stronger effects with clear trends towards enrichment and depletion were observed for the housekeeping and the tissue-specific gene sets (Supp.Fig. B12). As expected for housekeeping genes, the most notable enrichments with CLES values higher than 0.6 were predominantly found on transitions between activating states covering the promoter region and the gene body (Fig. 3A, Fig. 4), while strong depletions were mainly limited to transitions involving repressive and empty states, emphasizing the continuous expression of housekeeping genes throughout differentiation. Interestingly, with the maturation of ESC-derived to bone marrow-derived cell types of the ECL, we observed an enrichment of transitions from empty to transcribed states (ActGene and TxGene). In contrast, transitions into partially activated promoter states (PrimProm, PeriProm, InterEnh) were depleted (Fig. 3B). Such enrichments were not observed in the more specialized cell types of the MCL data (Supp.Fig. B12), indicating that the decoration of housekeeping genes with activating marks is strengthened during early differentiation stages and maintained thereafter.

In contrast to housekeeping genes, drastic state transitions, like empty to activating or repressive to activating were more prevalent in the tissue-specific gene sets, especially in the ECL, which covers more distant developmental stages (Supp.Fig. B12). For mesenchymal genes, the transitions into activating states were primarily enriched with the differentiation into eMSC and bmMSC, while the epigenetic activation of chondrogenic genes becomes more pronounced with the differentiation into chondrogenic cells (bmCC) (Fig. 3B). In contrast, in the MCL data transitions from repressed and empty into activating states were rarely detected while transitions between various activating states were more defined, especially on chondrogenic genes (Supp.Fig. B12).

One striking difference between housekeeping and tissue-specific mesenchymal and chondrogenic genes was the behavior of the RepActPro state, which carries a combination of several activating marks and the repressive H3K27me3. While the persistence of this state (self-transition of RepActPro to RepActPro) was depleted in the continuously expressed housekeeping genes, it was slightly enriched on mesenchymal and strongly enriched on chondrogenic genes (Fig. 4). Furthermore, in the tissue-specific genes, we also detected transitions from the RepActPro state into activating states (ActGene and ActProm) and vice versa, indicating a regulatory function of such transitions.

Specifically, in the ECL dataset, we found an enrichment of transitions from the RepActPro state into the activating states (ActGene and ActProm) on mesenchymal and chondrogenic genes. On mesenchymal genes, we observed small but directed effects from ESC to the more differentiated bmMSC, which are expected to express mesenchymal genes. On chondrogenic genes, the enrichment of this transition is more prominent and also clearly directed, with the effect size increasing with the differentiation from ESC to bmCC (Fig. 4). Importantly, the reverse transition, the gain of H3K27me3 on active promoters to form the RepActPro state, is not detectable in the ECL data.

The opposite was true for the MCL data, in which the RepActPro state rarely switched into activating promoter states. Instead, we found an enrichment of transitions from the activating promoter states into the RepActPro state on mesenchymal and chondrogenic genes. Importantly, although the effect sizes were relatively weak, the effect was clearly directed. On mesenchymal genes, the transition became more pronounced with increasing developmental distance from MSC to PC and MSC to HC, reflecting the repression of mesenchymal genes in mature PC and HC. On chondrogenic genes, the transition was mainly found with the differentiation into HC indicating that a subset of chondrogenic genes was downregulated with the onset of hypertrophy (Fig. 4).

To test the hypothesized critical association of the RepActPro state and downregulated gene expression, we used the extensive published knowledge about gene expression changes during chondrogenesis and inspected the genes undergoing the transition into the RepActPro state in detail. For the mesenchymal genes, this transition was detected on genes like Pdgfra, Itga3, and Fzd1 (Supp.Fig. B13A), which are downregulated upon the commitment to the chondrogenic fate [6, 26, 34]. The chondrogenic genes were split into two subsets: One subset showed the gain of H3K27me3 from MSC to PC and HC and included genes like Tgfb1, Hoxa11, Shox2, Wnt10b, and Msx2, which are closely associated with the initiation of the chondrogenic fate in MSC, but are downregulated upon its establishment [7, 39, 47, 57, 72] (Supp.Fig. B13B, Supp.Fig. B7). In the second subset, the transition occurred between PC and HC. This subset included genes like Col2a1, Col27a1, Runx3, Pth1r, Wnt5b, Rflna, and Acan, which are expressed in PC but downregulated in HC [27, 37, 38, 43, 65, 73, 74] (Supp.Fig. B13C, Supp.Fig. B7). Together these data strongly support a model in which the addition and removal of H3K27me3 on promoters marked as active are tightly associated with alterations in gene expression, thereby contributing to the control of cell lineage differentiation.

## Discussion

In our analyses, we used ChIP-Seq datasets acquired in different laboratories. On first inspection, we detected major differences in the overall coverage of distinct histone marks. Most strikingly, in the ECL dataset, ESC and eMSC display a generally low decoration of the investigated marks, while in the MCL dataset, we found an increased coverage of H3K27me3 in MSC. One problem of such analyses is that technical differences in data acquisition can lead to overall differences in coverage or specificity. If an attribute, like the global histone modification level, differs between datasets one has to determine if such differences are specific to the differentiation process, represent general features of the cell type, or are due to the respective experimental setting. Another challenge is the supposedly low proportion of genes that change their expression and thus their epigenetic state with cellular differentiation, especially if direct differentiation steps are investigated. To overcome both problems, we focused on the analysis of tissue-specific genes and compared their epigenetic coverage to that of the background. If the relative coverage is similar in the investigated cell types, differences in the overall coverage of a histone modification in single datasets are likely not related to their differentiation but rather to technical differences in data acquisition. In contrast, if the relative coverage on tissue-specific genes deviates from the background the respective modification is likely linked to a cell type-specific expression pattern.

Using this principle, drastic differences in histone coverage, like the increased prevalence of H3K27me3 and its associated SilGene state in MSC, vanished. This strongly indicates that the increase of H3K27me3 in MSC is not linked to the expression of tissue-specific genes but is rather caused by the different laboratory origin. In contrast, the relative approach detected the expected increased coverage of activating states and a reduced decoration with repressive chromatin states on housekeeping genes, illustrating that this contextual strategy indeed makes biological interpretation of data with technical differences possible.

Our study involved independent analyses of data from humans and mice. Consequently, we can formally not exclude that differences in state emission between the ECL and MCL datasets reflect species-specific differences. However, the 15-state ChromHMM models included 13 states with a highly similar emission in both datasets. The respective two different states had low coverage and were associated with promoter regions, which showed a high fluctuation of states with overlapping emissions in both models. For the remaining 13 states, the humanized MCL model closely mimicked the murine model, strongly indicating that the differences in the state emission are not critical for the identity of the state. This is further supported by the slight differences in state emission of the human ECL and ESC-only model. Similarly, in a previously published murine PC/HC-only model [71], the RepActPro state included only marginal H3K9me3 emission. Consistent with our data, ChromHMM models derived from similar histone marks detected bivalent states with varying coverage of promoter marks [24]. Furthermore, a recent comparison of a comprehensive murine ChromHMM model based on 900 epigenomes from different cell types with the corresponding human model showed only small differences between the human and murine epigenome [66]. Taken together, these results indicate that differences in emission reflect the composition of cell types rather than species-specific differences.

To investigate how changes between epigenetic states are associated with tissue differentiation we have developed BATH, a Bayesian model that allows us to study the transitions of chromatin states in the course of cellular differentiation. Its design enables the systematic analysis of every possible transition of chromatin states, irrespective of the experimental origin of the data. In such systematic analyses, the number of outcome variables grows quickly with the number of cell types and states. For example, in the case of the MCL dataset, there are 6 possible combinations of samples with 15 times 15 possible state transitions, resulting in 1350 unique transitions for each group of genes. Since small random enrichments and depletions will inevitably occur for any given group, the risk of overinterpreting random fluctuations as meaningful effects is high.

To assess the scale that we can expect for this type of noise, we initially analyzed subsets of random genes. As expected, we observed no systematic enrichment or depletion pattern and an effect size (CLES) close to 0.5, indicating no systematic enrichment or depletion. In contrast, the housekeeping and tissue-specific genes showed much larger effect sizes for distinct transitions. Generally, we observed shifts towards transitional enrichment of activating states, while depletions are mostly limited to empty and repressive states. This general pattern is particularly pronounced in the set of housekeeping genes, with multiple transitions between activating states well above a CLES of 0.6.

Variations of this general pattern often highlight cellular characteristics or relationships. For example, as described above, we observed two significant global differences between the datasets, the increased H3K27me3 levels in murine MSC and the lower coverage of all histone marks in human ESC and ESC-derived MSC. While the drastic difference in H3K27me3 vanished if analyzed relative to the background, the lineage-specific modification pattern of ESC and ESC-derived MSC is maintained in the transition analysis. These results are in line with several studies that describe the biological heterogeneity of MSC obtained from different sources [13, 22, 32, 62] and reinforce the need to carefully characterize MSC not only at the molecular but also at the epigenetic level before using them for therapeutic or regenerative purposes.

Studying the individual transitions in more detail revealed two distinct modes. The first type includes non-directional transitions, with similarly large effect sizes within a cell type (e.g. MSC to MSC) and between cell types (e.g. MSC to PC). The second type of transition shows a directed pattern, like a continuous increase or decrease over sequential differentiation stages. The non-directional transition type is best exemplified by transitions between various activating states on the promoter or transcribed gene body. The undirected pattern implies that the gain or loss of single histone marks in these states is not indicative of the differentiation, but merely represents the high fluctuation of the respective histone modifications, forming a dynamic equilibrium. In accordance with their stable, differentiation-independent expression, such undirected transitions, with high CLES values, are found most prominently in housekeeping genes.

For mesenchymal and chondrogenic genes these non-directional transitions between activating states are less pronounced, and we see a more diverse set of transitions. This is most obvious for the wide differentiation path of the ECL dataset, in which we observe the enrichment of directed transitions from repressed or empty into various activating states and thus a clear correlation between cellular differentiation and effect strength. For mesenchymal genes, the effect size often peaks for the transition of ESC into eMSC and bmMSC, while for chondrogenic genes the effects peak for transitions into bmCC. In contrast, in the MCL data, drastic directed changes from repressed or empty into activating states were not detected. This is in accordance with the sequential initiation of gene expression during lineage specification, while at later differentiation stages, the expression-activating decoration has already been initiated [50, 63].

One of the most pronounced directed effects is observed for the transition from the RepActPro state into the ActGene state on mesenchymal and chondrogenic genes. The RepActPro state can be regarded as a subtype of the classical bivalent state, characterized by the combination of H3K27me3 and H3K4me3. This state has been identified in ESC and is thought to poise lineage-specific gene expression. Subsequently, expression is initiated by loss of the repressive mark [4, 8, 24, 33, 62]. Intriguingly, we did not detect the classical bivalent state in the ChromHMM model of either dataset, although ESC were included in the ECL data. It was however detected if a 15-state ChromHMM model was generated based on only ESC data, indicating that the classical bivalent state transiently occurs in ESC and is likely linked to high pluripotency in these cells. As even under these conditions the RepActPro state was also detected, one might speculate that the classical bivalent state is transformed into a more complex combination of active and repressive marks with the onset of differentiation. Interestingly, the bivalent state can also be detected in the other cell types if the ESC-specific ChromHMM model is used to define states. The sequential addition of activating marks to still repressed promoters might thus represent a general mechanism allowing the fast switch to gene expression by removing H3K27me3.

In the MCL dataset, we noted the opposite behavior, the formation of the RepActPro state by the gain of H3K27me3 on active promoters. In the mesenchymal gene set, this transition is strictly directed from MSC to PC and HC and was found on genes that are required to initiate the chondrogenic fate but are turned off at mature stages. Similarly, on chondrogenic genes, this transition was detected from MSC to PC and HC on genes associated with the initiation of chondrocyte differentiation [10, 23, 48, 54, 57], while from PC to HC the transition marked genes that are downregulated at later hypertrophic stages [18, 27, 43, 73, 74]. These results corroborate previous findings on the tight association of the addition of H3K27me3 to active promotors of genes that become repressed in the course of chondrocyte differentiation [71] and strongly point to an instructive role in gene repression.

## Conclusion

Taken together, our results indicate that the addition of H3K27me3 to active promoters is strongly associated with cell lineage progression. Furthermore, it seems to be more widely used than previously described, demarcating genes poised for lineage-specific expression in ESC but also defining the initiation of gene repression of lineage-specific genes during later cellular differentiation. In line with these findings, the bivalent state has been associated with several differentiated tissues [5, 16, 30, 52, 56] (reviewed in Blanco et al. [9]). Combined with the findings presented here, this challenges the notion that the characteristic bivalent combination of H3K4me3 and H3K27me3 is specific to ESC. Instead, the interplay of these activating and repressive marks, likely in more complex forms than previously thought, appears to be a general mechanism for initiating expression switches linked to cellular differentiation.

## Supplementary Information

Supplementary file 1. Fig. B1 Illustration of the analytical workflow.: (A) Overview of the workflow illustrating the relationship between consensus and individual ChIP-Seq datasets and their processing for transition analysis. (B) Diagram depicting the exemplary transition from the ActProm state (purple cell) to the RepActPro state (beige cell). Transition probabilities were determined for the investigated groups of genes and the background. The CLES value reflects the transition probability of a gene for a given group (a or b) relative to the background. CLES > 0.5 corresponds to an enrichment, while CLES < 0.5 corresponds to a depletion.

Supplementary file 2. Fig. B2 Comparison of CLES values for the group size of N=1 and N=100.: Each data point represents the effect size for a given transition with N=1 and N=100 (see methods). For the majority of transitions, there is a direct correlation between both values, illustrating only minor contributions to the variance.

Supplementary file 3. Fig. B3 Jaccard indices between the individual datasets for the 6 histone modifications of ECL (ESC, eMSC, bmMSC, and bmCC) and MCL (MSC, PC, and HC) data, depicting similarities and differences between the peak-called data.: Both datasets show a high similarity of any given mark between the analyzed cell types with four main clusters comprised of activating promoter marks (H3K4me3, H3K9ac, and H3K27ac), the activating gene body mark H3K36me3, and the repressive marks H3K9me3 and H3K27me3. (A) In the ECL dataset, each mark correlates strongest with the same mark across the cell types, with the bone marrow-derived cell types showing the highest correlation with each other. (B) In the MCL dataset, the repressive marks of MSC show strong correlations with each other, but significantly weaker correlations with the same marks in other cell types.

Supplementary file 4. Fig. B4 Analytical metrics as reported by ChromHMM for the consensus data of the ECL (A-C) and MCL (D-F) data.: (A and D) Correlating the model’s number of states and the estimated log-likelihood shows saturation around 15 states. (B and E) Fold enrichment of the individual states on selected genomic regions (column-wise scaled to one. Due to the high prevalence of the Emp state in the genome, the genome section is set to a different color scale (red)). (C and F) Neighborhood enrichment of the 15 states surrounding the anchor points (transcriptional starting and end site; TSS and TES, respectively). White indicates no enrichment and dark blue is the strongest enrichment for the particular combination of cell type and anchor point.

Supplementary file 5. Fig. B5 Comparison of state emissions and coverage.: The left side of the individual figures shows the emission probability of the ChromHMM model fitted with the consensus data. The right side shows the corresponding genomic coverage of a given state for each replicate. (A) and (C) represent the full dataset of the ECL and MCL, respectively. (B) represents the ChromHMM model fitted only with the consensus data of the ESC samples. The two states highlighted with an arrow (Bivalent and RepActPro) show the lowest correlation (euclidean distance) to any other state in the ECL. The color of the row names categorizes the states into four groups (green: activating, orange: repressed-active, red: repressive, and grey: empty).

Supplementary file 6. Fig. B6 Jaccard Index between Murine and Humanized ChromHMM Models.: Both ChromHMM models show a high similarity of the individual states, including states with slight emission differences like the RepActProm state. The two murine states with vastly different emissions in the human model (FlnkTSS and ProxEnh) do not have clear counter parts,thus weakly colocating with different promoter and enhancer states of the humanized dataset. For a more detailed view of the differences on genes of interest, see Supp. Fig. B7.

Supplementary file 7. Fig. B7 Chromatin state coverage on lineage-defining genes.: The murine and humanized ChromHMM states were uploaded to the UCSC genome browser. Chondrogenic genes with the highest number of transitions (Supp.Fig. B13) are depicted (Tgfb1 (A), Shox2 (B), Osr1 (C) and Col27a1 (D)). Transitions from the ActGene (dark green) or ActProm (light green) to the RepActPro state (orange) were mainly detected in the promoter region. No significant differences were detected between the murine and the humanized model.

Supplementary file 8. Fig. B8 Comparison of the coverage of ChromHMM states on gene sets of interest to background genes for the ECL (A) and MCL (B).: Coverage in base pairs (bp) of a given ChromHMM state (row) on a given gene set (column) plotted against the coverage on all genes (background). The black line (slope = [length of all background genes] / [length of all genes within a set], intercept = 0) delineates an equal proportional coverage between the background and the gene set of interest. See also Fig. 2.

Supplementary file 9. Fig. B9 Convergence diagnostic for the Bayesian transition model.: Models for ECL and MCL have well-mixed chains with ***R***-values close to 1, below the recommended upper bound of about 1.05 [61].

Supplementary file 10. Fig. B10 Posterior predictive checks for the Bayesian transition models for ECL (A-B) and MCL (C-D).: Contrasting the number of genes with a given transition (x-axes) against the average expected number of genes based on the model’s parameter estimates (y-axes). Each data point represents a specific tissue pair (indicated by color and shape) and gene set of interest. The variation along the x-axis reflects differences between replicates, grouped by a common y-value, which represents the model’s estimate of the average expected number of genes. Due to the vastly different scale, the data are split into measurements and estimates of the background (A, C) and measurements and estimates of the gene sets (B, D).The gray line indicates a perfect match between the measurements and the average estimates. For an overview of the model’s performance, see Supp. Fig. B11.

Supplementary file 11. Fig. B11 Summary of Posterior predictive checks for the Bayesian transition models. The mean difference between the estimated and observed number of genes (y-axes) is plotted against the observed gene count (x-axes), for ECL (A) and MCL (B). Each datapoint represents a specific combination of gene set, tissue pair, and state transition. The majority of combinations show a difference very close to 0. Very small and large gene counts exhibit systematic over- or underrepresentation compared to the observed data, respectively. This is expected, as the model is designed to be more conservative with extreme values. When the observed data are less reliable (e.g. fewer replicates, as seen for MSC to MSC and HC to HC, with only two replicates each), the model becomes more cautious with extreme values, resulting in an increased difference. For a more detailed illustration of the PPC, see Supp. Fig. B10.

Supplementary file 12. Fig. B12 CLES values calculated for each transitional enrichment and depletion (CLES) for every state and cell type combination for each set of genes and type of dataset (ECL or MCL).: The rows indicate the originating and the columns the target states of the respective cell types. Colored axes classify the states as empty (gray), repressive (red), repressed-active (orange), and activating (green). The blue and red squares demarcate the transitions highlighted in Fig. 3 and Fig. 4, respectively.

Supplementary file 13. Fig. B13 Occurrence rate for the gain of the repressive mark H3K27me3 on activating states in mesenchymal and chondrogenic genes of the MCL data.: In the word cloud, the size and color indicate in how many combinations of samples any given gene shows the transition from the ActProm or ActGene to the RepActPro state. Numbers show the combined occurrence of both transitions on each gene. (A) Transitions from MSC to PC on mesenchymal genes. With 2 and 3 replicates respectively, this leads to a maximum of 12 possible occurrences for a given gene (6 combinations of replicates for each of the 2 transitions). (B) Transitions from MSC to PC or HC on chondrogenic genes. With 2, 3, and 2 replicates respectively, this leads to a maximum of 24 possible occurrences. (C) Transition from PC to HC on chondrogenic genes. With 3 and 2 replicates respectively, this leads to a maximum of 12 possible occurrences. See Supp. Tab. A3 for the data visualised in this figure.

Supplementary file 14. Table A1 SRA-IDs: Reference IDs of murine MSC data.

Supplementary file 15. Table A2 Sets of interest.: Genes contained in all sets of interest. This includes housekeeping genes, chondrogenic genes, mesenchymal genes, and all random control sets.

Supplementary file 16. Table A3 Occurrence rate for the gain of the repressive mark H3K27me3 on activating states in mesenchymal and chondrogenic genes of the MCL data.: The frequency column gives the number of transitions from the ActProm or ActGene to the RepActPro state for all genes in the set and all combinations of samples. The table includes three types of transitions. (A) Transitions from MSC to PC on mesenchymal genes. With 2 and 3 replicates respectively, this leads to a maximum of 12 possible occurrences for a given gene (6 combinations of replicates for each of the 2 transitions). (B) Transitions from MSC to PC or HC on chondrogenic genes. With 2, 3, and 2 replicates respectively, this leads to a maximum of 24 possible occurrences. (C) Transition from PC to HC on chondrogenic genes. With 3 and 2 replicates respectively, this leads to a maximum of 12 possible occurrences. For a visualization of the data see Supp. Fig. B13.

## Data Availability

Data is publicly available and was obtained via SRA and https://egg2.wustl.edu/roadmap/data.

## Code availability

The source code to download the initial data and reproduce the full analysis, is available via https://gitlab.com/kEks/bath
