# Supplementary figures and images for: Bath: a Bayesian approach to analyze epigenetic transitions reveals a dual role of H3K27me3 in chondrogenesis
Source: Epigenetics Chromatin. 2025 Jun 27;18:38. doi: 10.1186/s13072-025-00594-6 (PMC12203727; doi:10.1186/s13072-025-00594-6)

**A**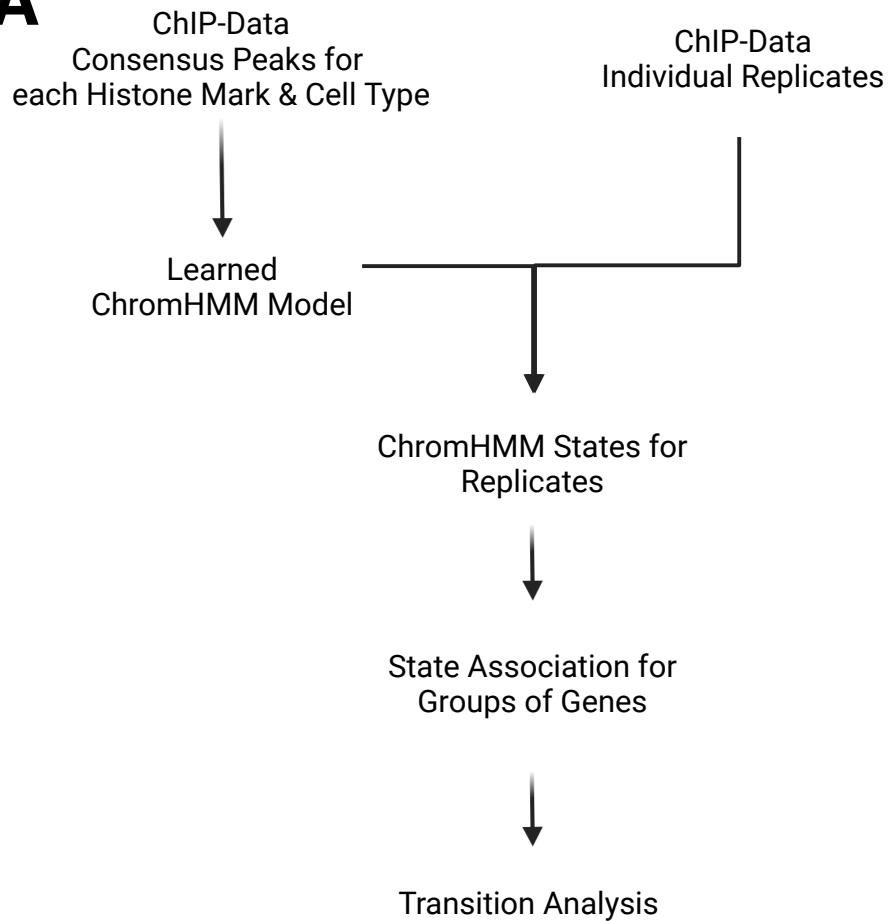**B**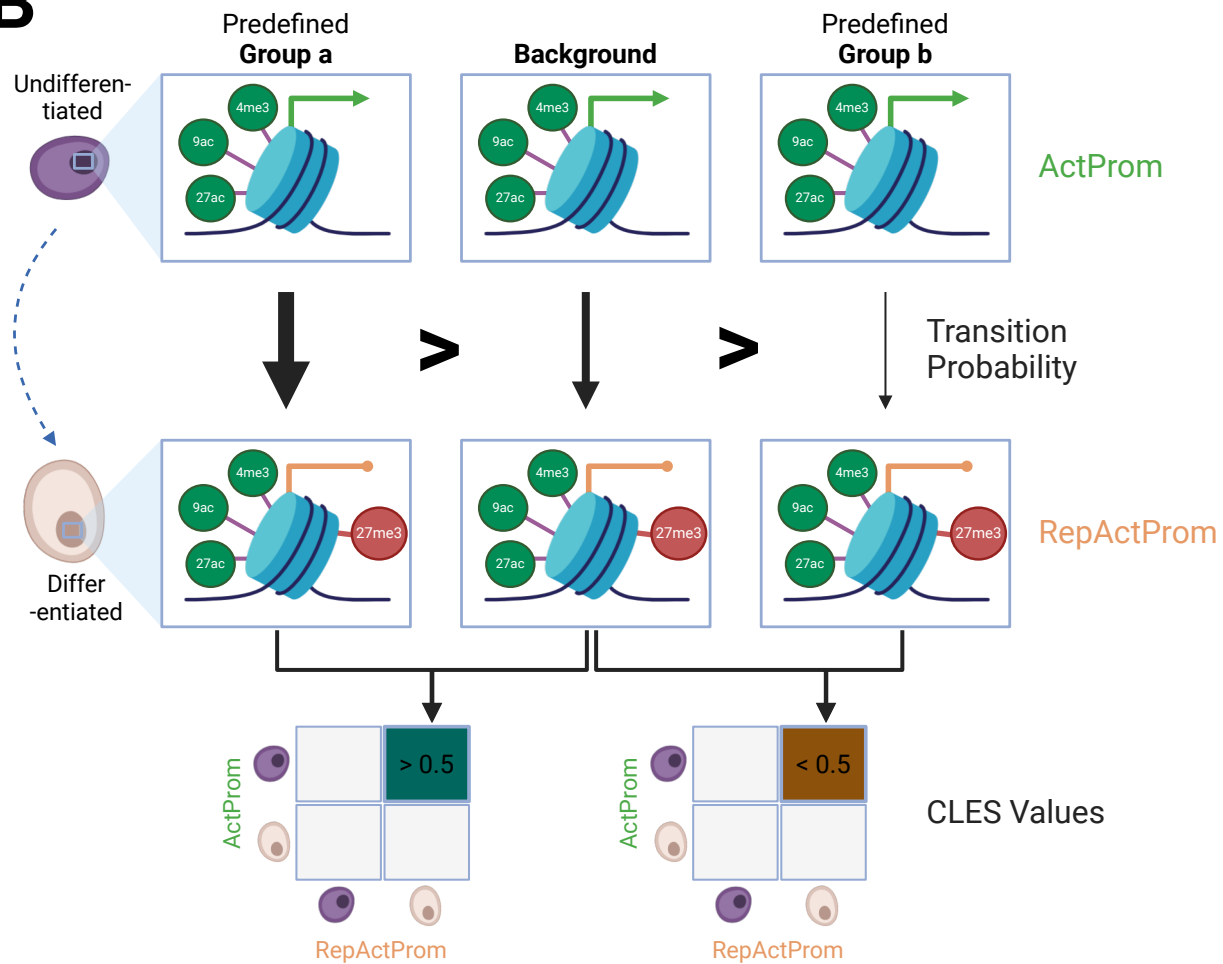

Supplement: Supplementary file 1 — Fig. B1 Illustration of the analytical workflow.: (A) Overview of the workflow illustrating the relationship between consensus and individual ChIP-Seq datasets and their processing for transition analysis. (B) Diagram depicting the exemplary transition from the ActProm state (purple cell) to the RepActPro state (beige cell). Transition probabilities were determined for the investigated groups of genes and the background. The CLES value reflects the transition probability of a gene for a given group (a or b) relative to the background. CLES > 0.5 corresponds to an enrichment, while CLES < 0.5 corresponds to a depletion. [file 13072_2025_594_MOESM1_ESM.pdf]

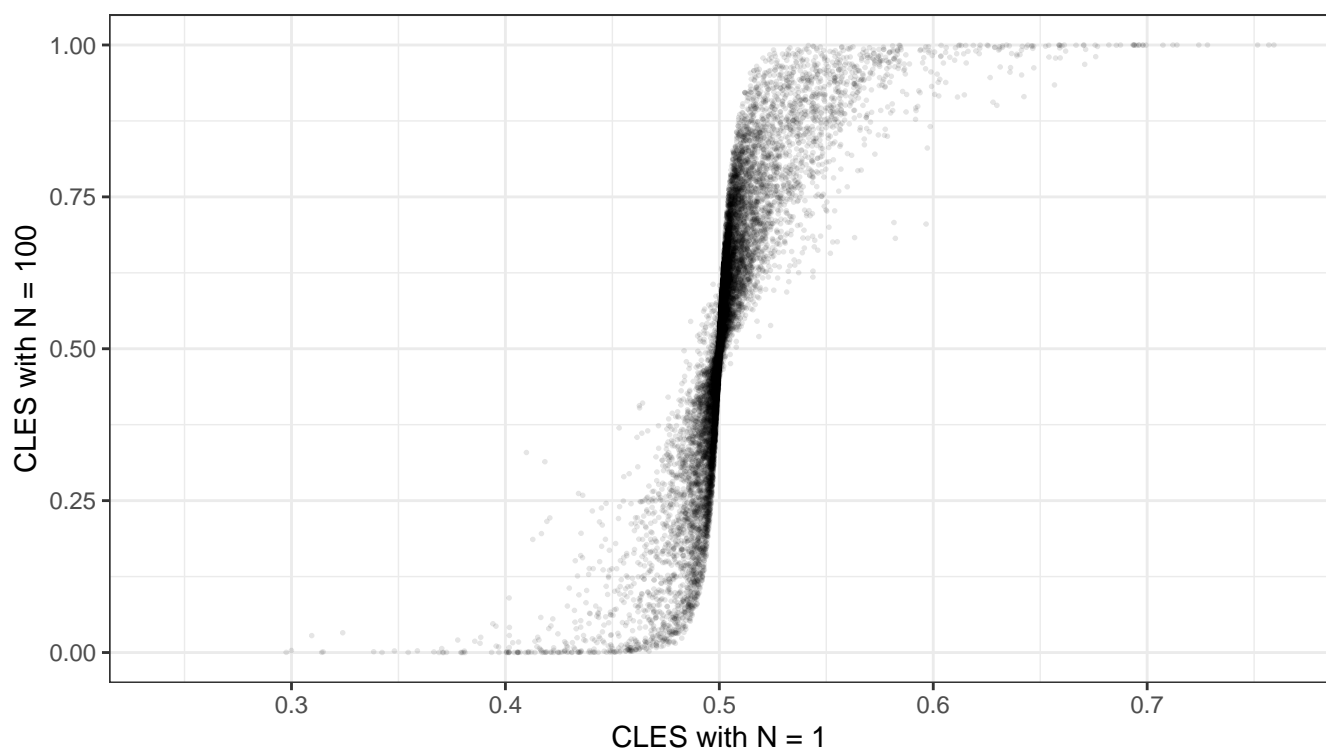

Supplement: Supplementary file 2 — Fig. B2 Comparison of CLES values for the group size of N=1 and N=100.: Each data point represents the effect size for a given transition with N=1 and N=100 (see methods). For the majority of transitions, there is a direct correlation between both values, illustrating only minor contributions to the variance. [file 13072_2025_594_MOESM2_ESM.pdf]

EC

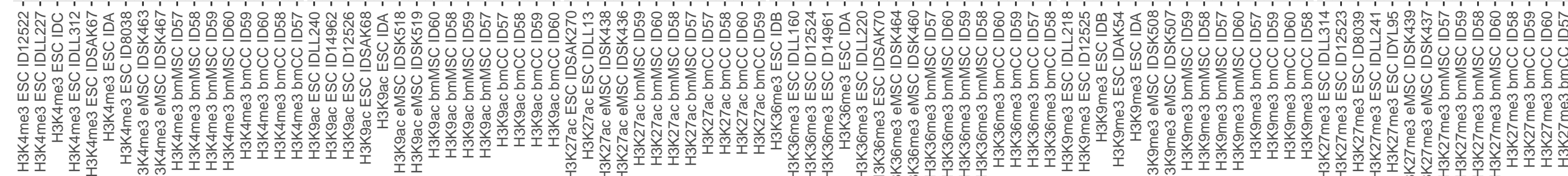

M

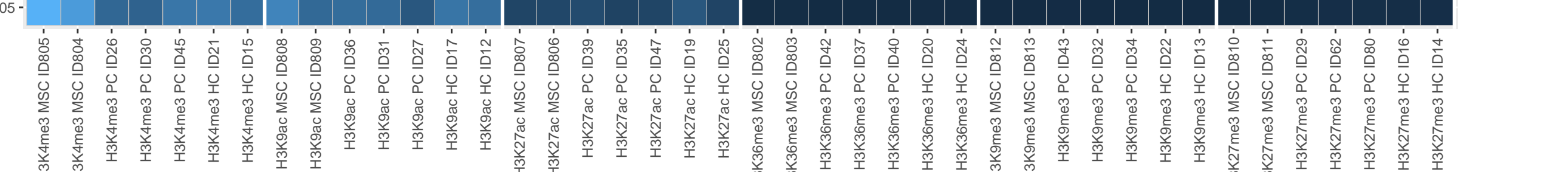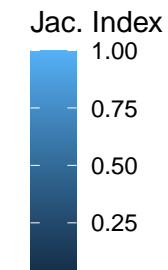

Supplement: Supplementary file 3 — Fig. B3 Jaccard indices between the individual datasets for the 6 histone modifications of ECL (ESC, eMSC, bmMSC, and bmCC) and MCL (MSC, PC, and HC) data, depicting similarities and differences between the peak-called data.: Both datasets show a high similarity of any given mark between the analyzed cell types with four main clusters comprised of activating promoter marks (H3K4me3, H3K9ac, and H3K27ac), the activating gene body mark H3K36me3, and the repressive marks H3K9me3 and H3K27me3. (A) In the ECL dataset, each mark correlates strongest with the same mark across the cell types, with the bone marrow-derived cell types showing the highest correlation with each other. (B) In the MCL dataset, the repressive marks of MSC show strong correlations with each other, but significantly weaker correlations with the same marks in other cell types. [file 13072_2025_594_MOESM3_ESM.pdf]

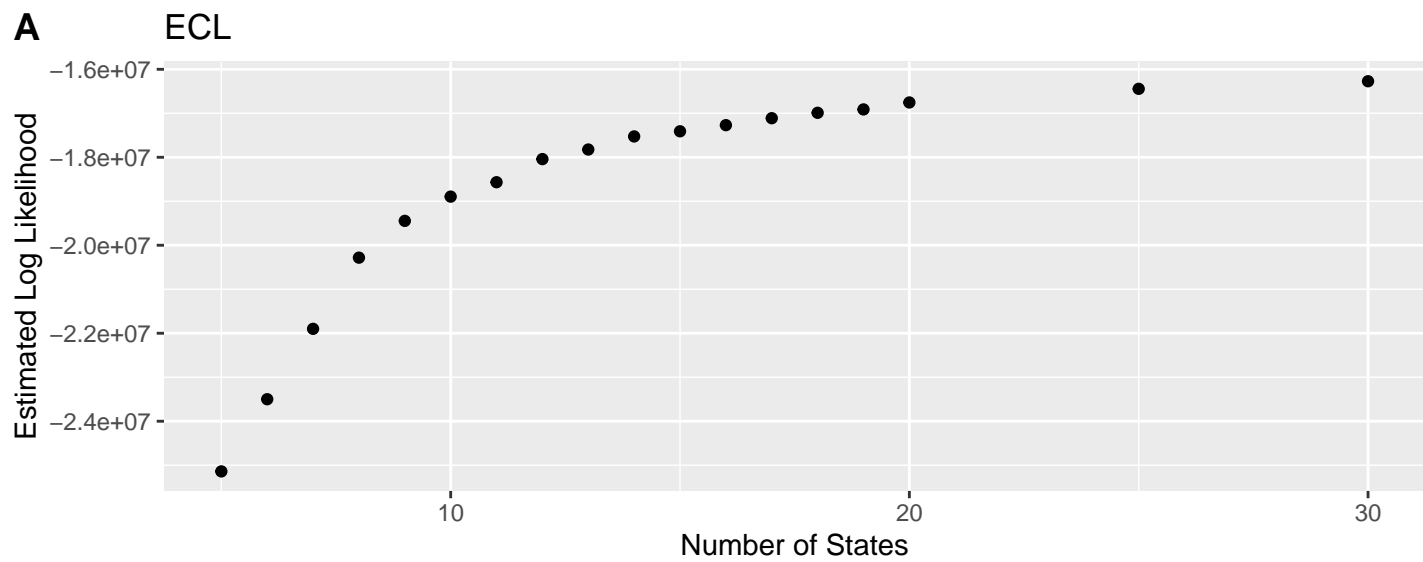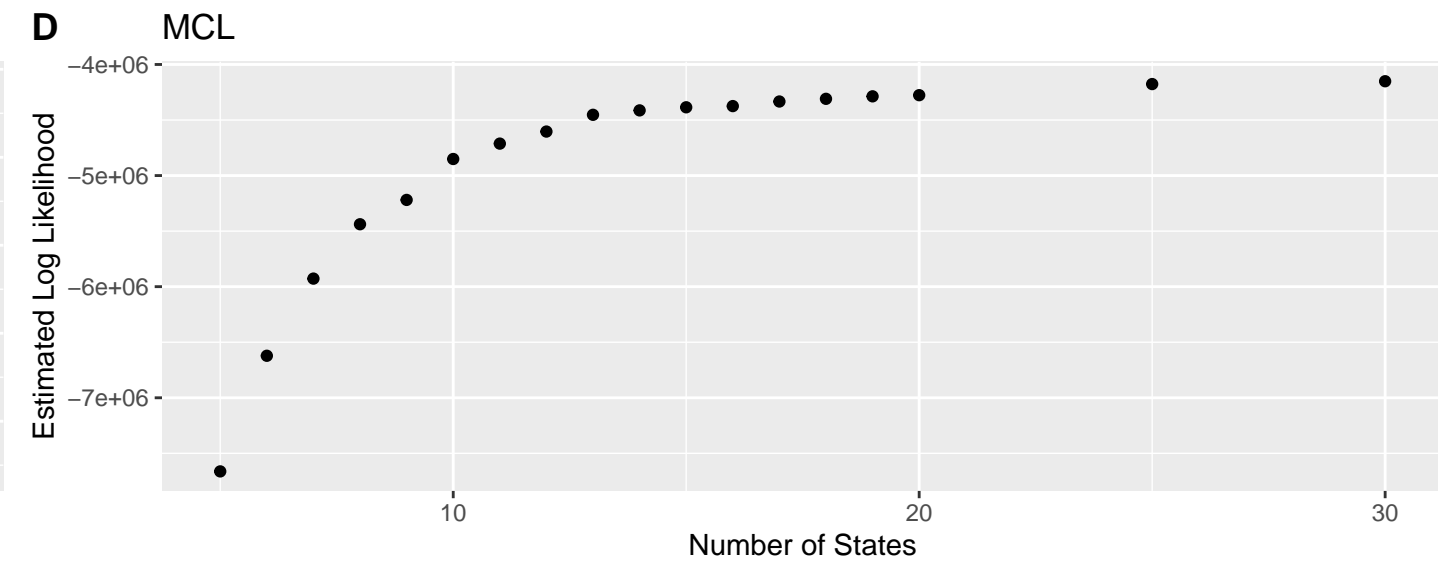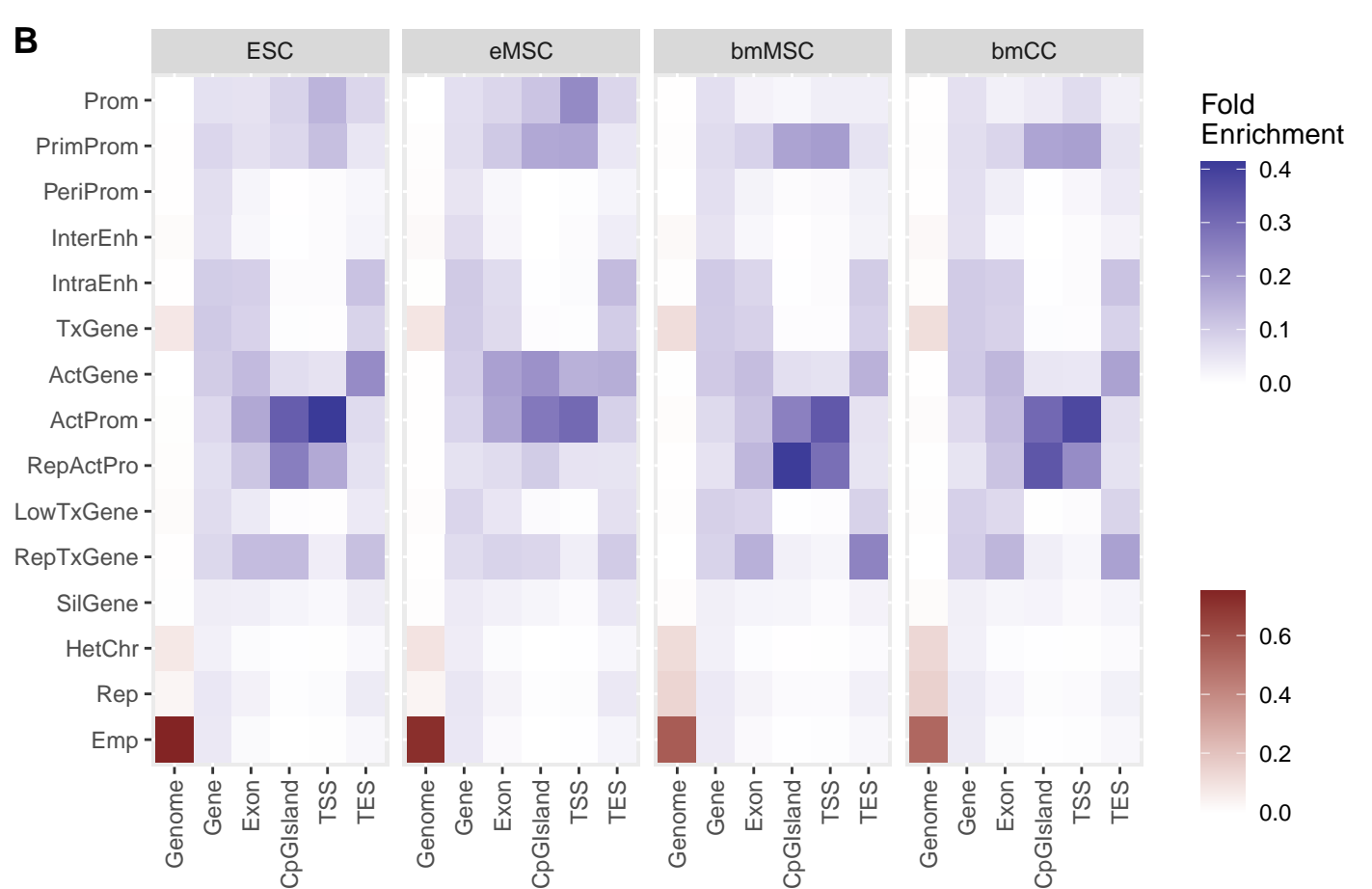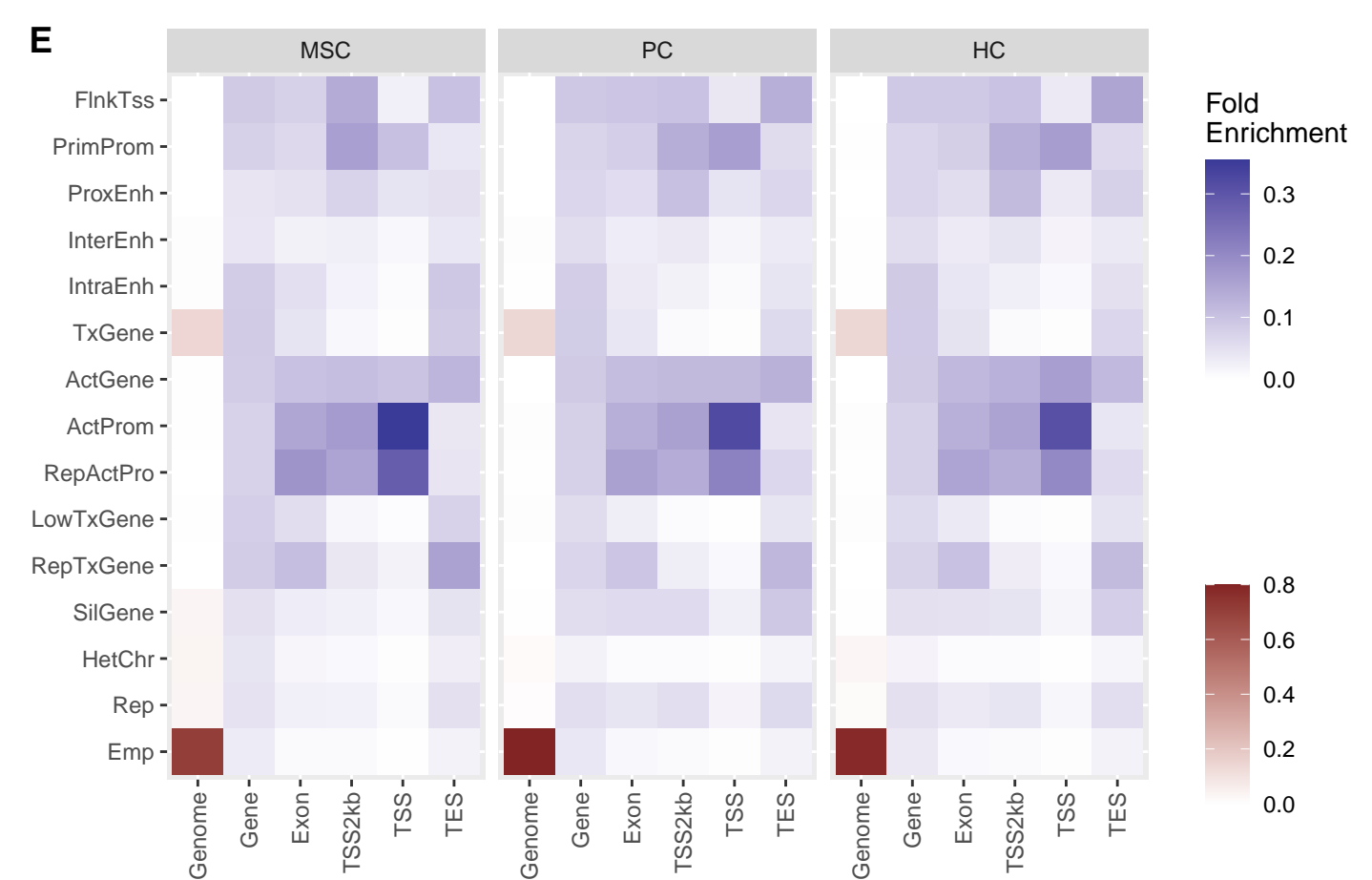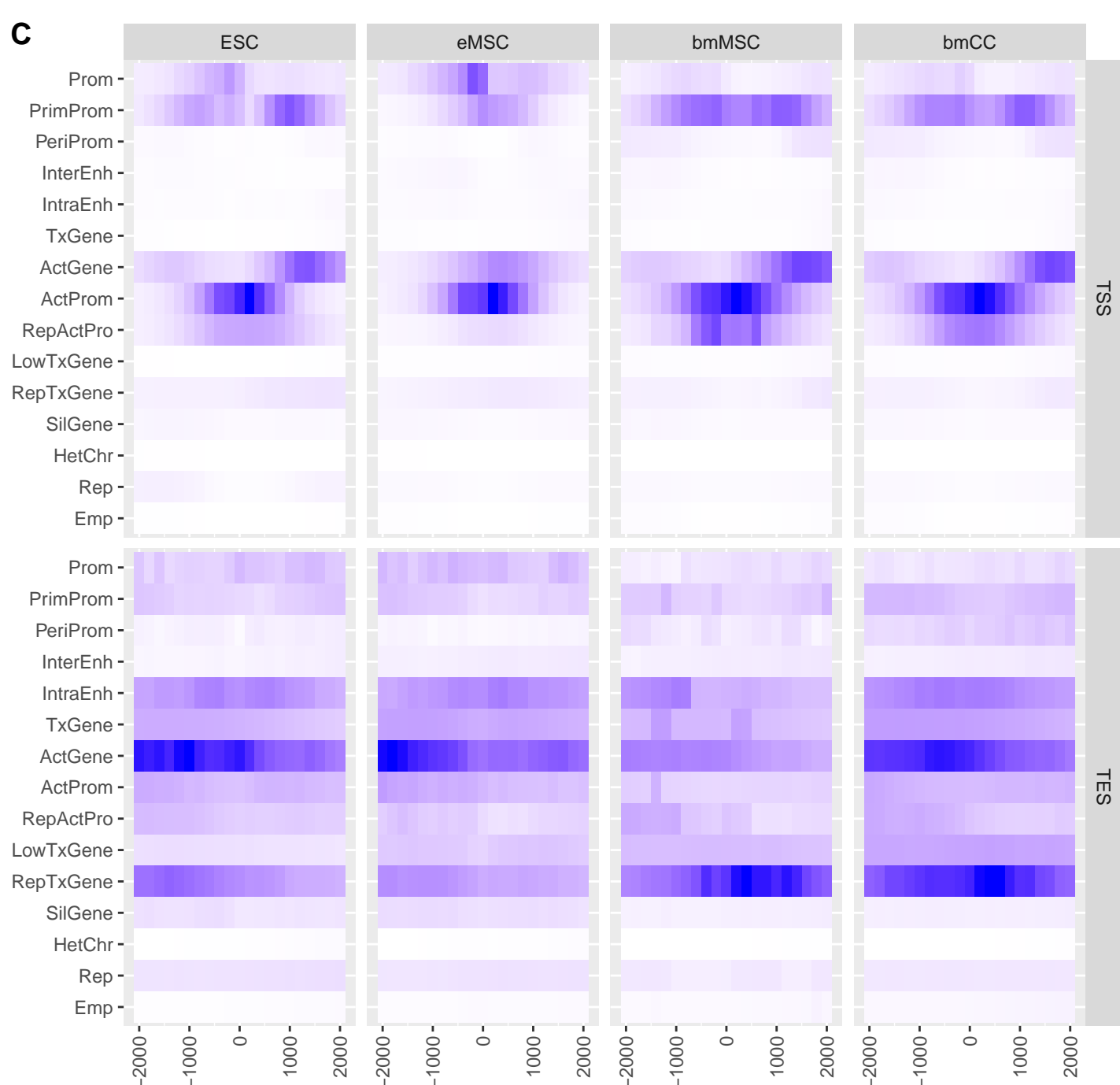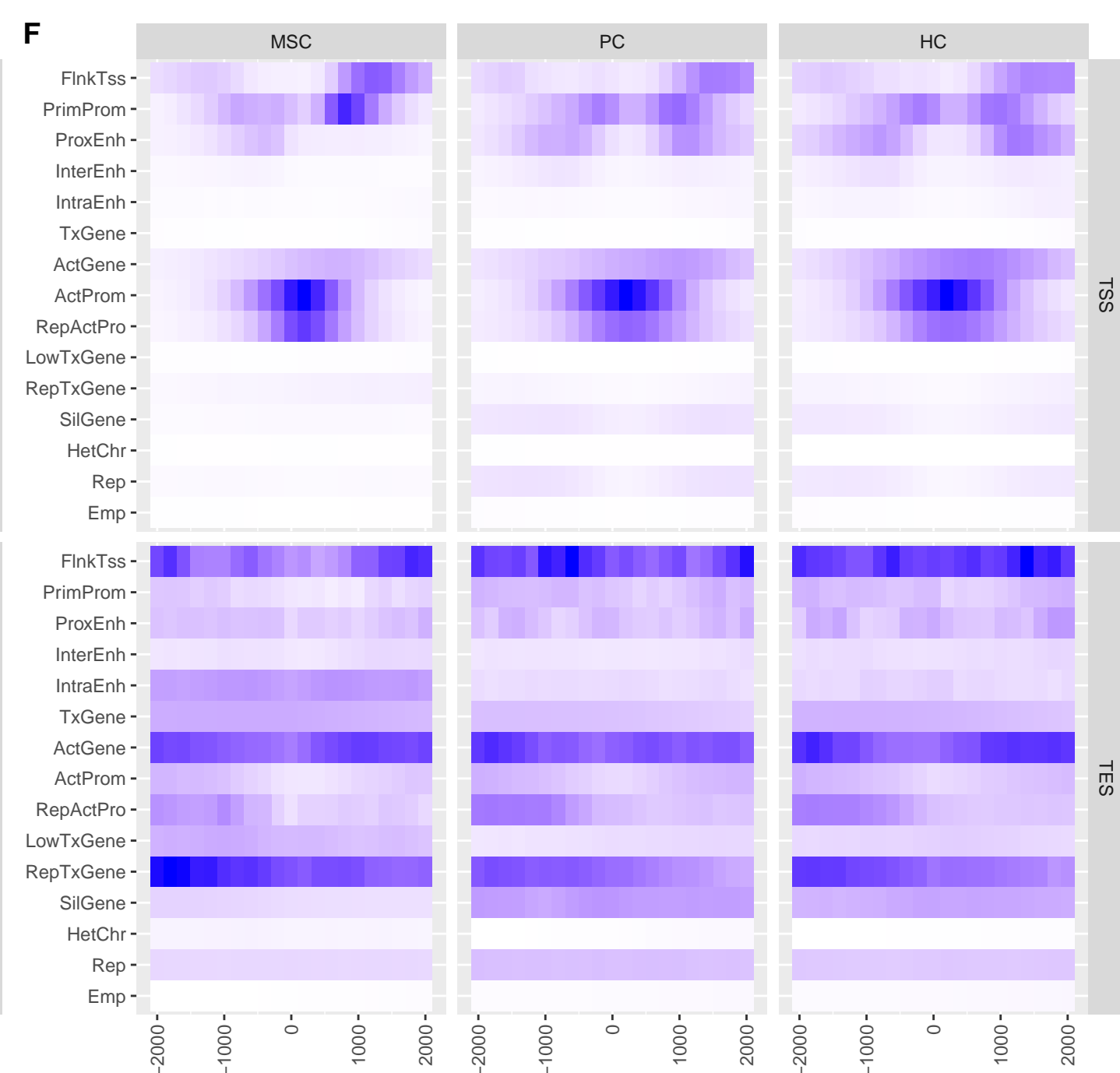

Supplement: Supplementary file 4 — Fig. B4 Analytical metrics as reported by ChromHMM for the consensus data of the ECL (A-C) and MCL (D-F) data.: (A and D) Correlating the model’s number of states and the estimated log-likelihood shows saturation around 15 states. (B and E) Fold enrichment of the individual states on selected genomic regions (column-wise scaled to one. Due to the high prevalence of the Emp state in the genome, the genome section is set to a different color scale (red)). (C and F) Neighborhood enrichment of the 15 states surrounding the anchor points (transcriptional starting and end site; TSS and TES, respectively). White indicates no enrichment and dark blue is the strongest enrichment for the particular combination of cell type and anchor point. [file 13072_2025_594_MOESM4_ESM.pdf]

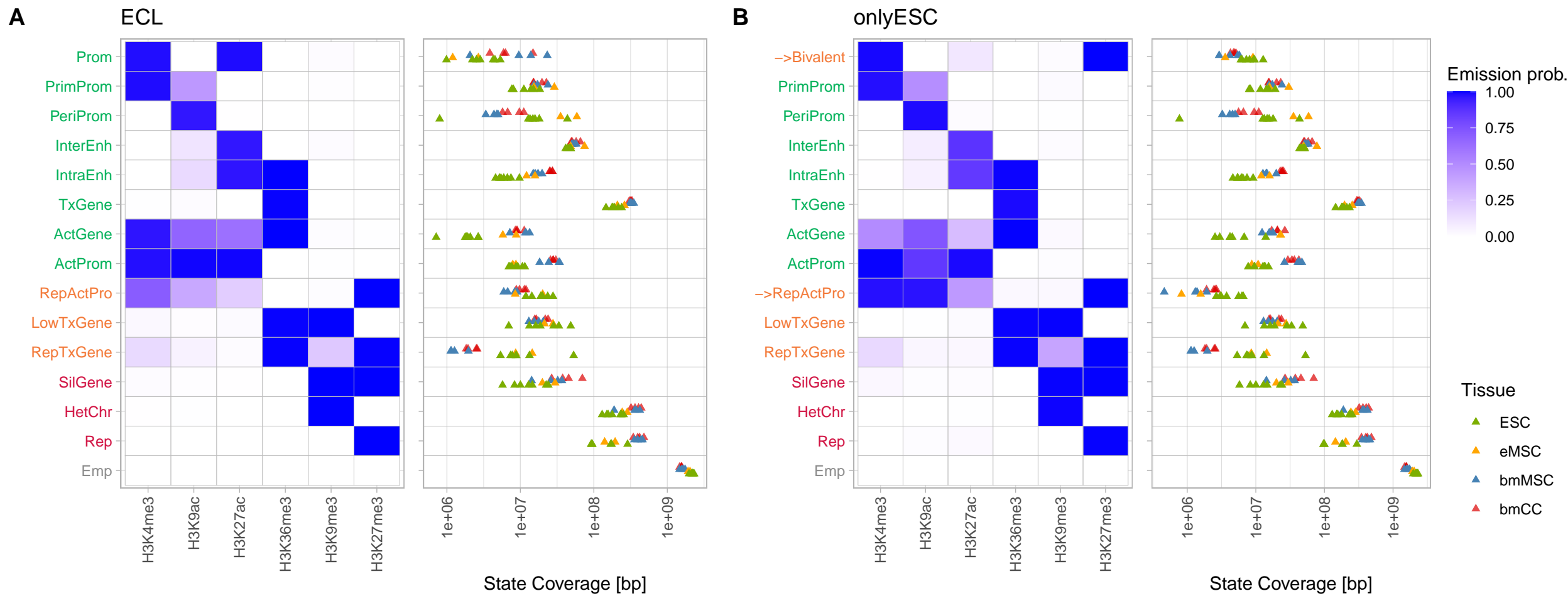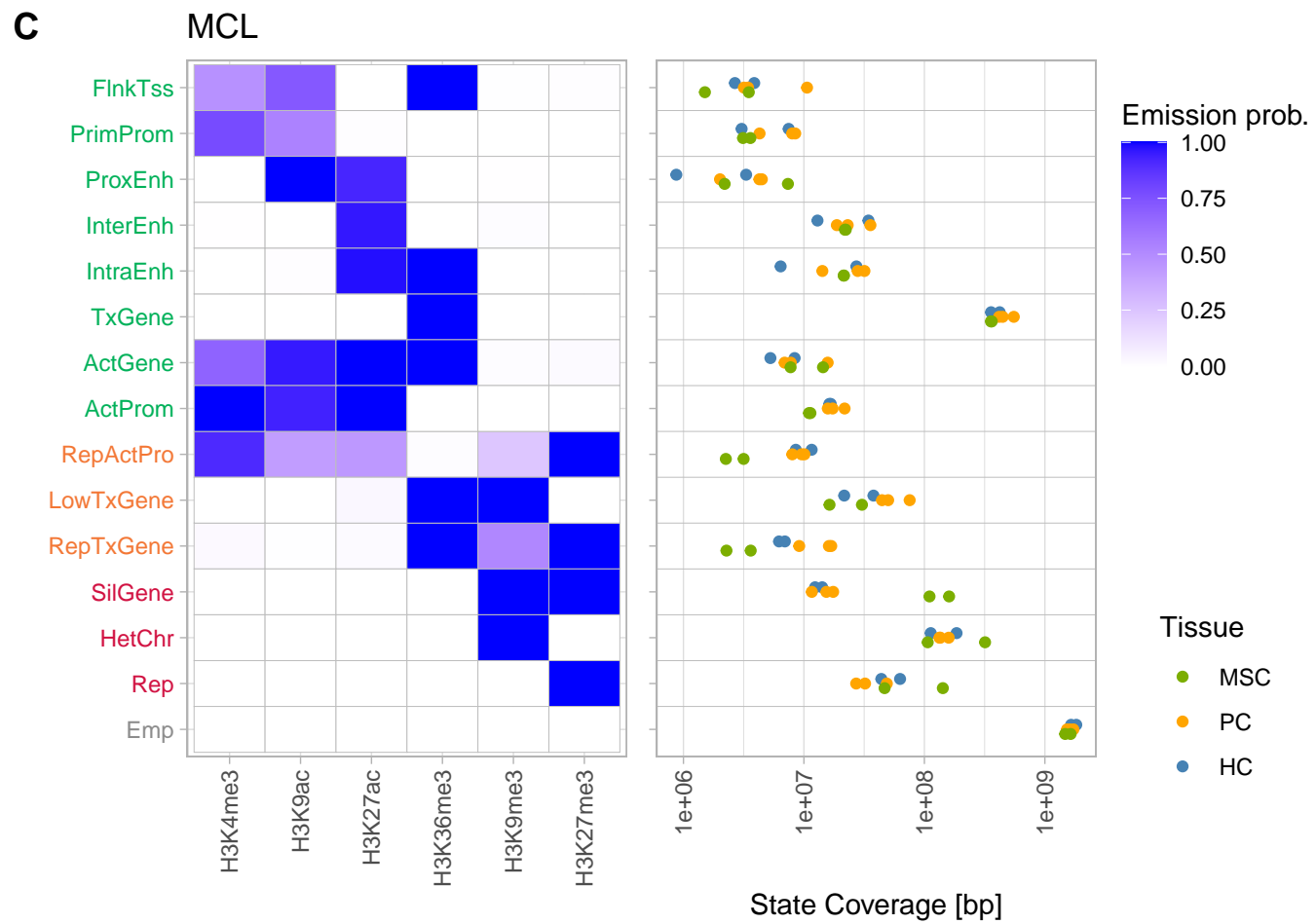

Supplement: Supplementary file 5 — Fig. B5 Comparison of state emissions and coverage.: The left side of the individual figures shows the emission probability of the ChromHMM model fitted with the consensus data. The right side shows the corresponding genomic coverage of a given state for each replicate. (A) and (C) represent the full dataset of the ECL and MCL, respectively. (B) represents the ChromHMM model fitted only with the consensus data of the ESC samples. The two states highlighted with an arrow (Bivalent and RepActPro) show the lowest correlation (euclidean distance) to any other state in the ECL. The color of the row names categorizes the states into four groups (green: activating, orange: repressed-active, red: repressive, and grey: empty). [file 13072_2025_594_MOESM5_ESM.pdf]

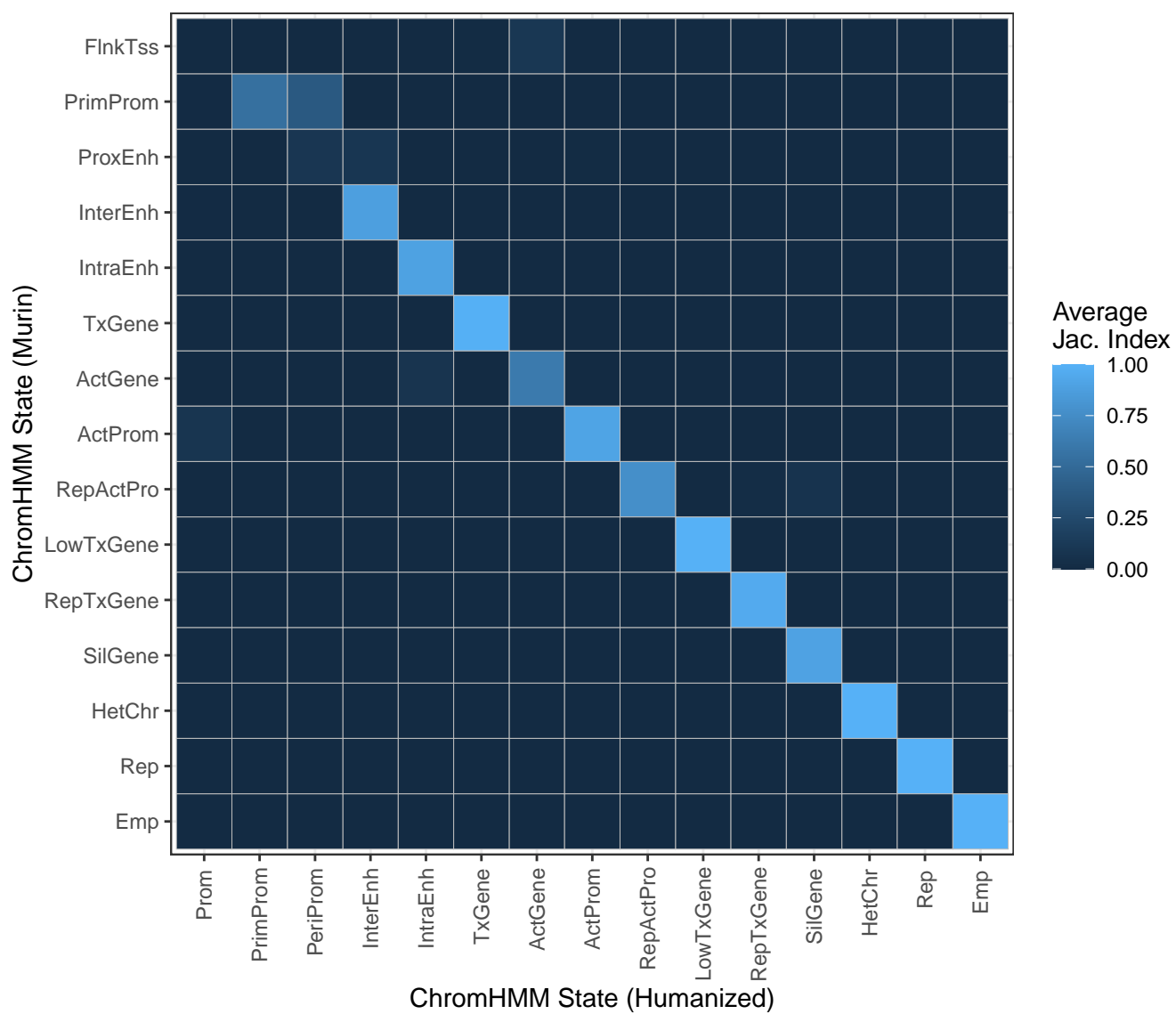

Supplement: Supplementary file 6 — Fig. B6 Jaccard Index between Murine and Humanized ChromHMM Models.: Both ChromHMM models show a high similarity of the individual states, including states with slight emission differences like the RepActProm state. The two murine states with vastly different emissions in the human model (FlnkTSS and ProxEnh) do not have clear counter parts,thus weakly colocating with different promoter and enhancer states of the humanized dataset. For a more detailed view of the differences on genes of interest, see Supp. Fig. B7. [file 13072_2025_594_MOESM6_ESM.pdf]

# A

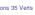

# B

**C**

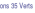

## D

Supplement: Supplementary file 7 — Fig. B7 Chromatin state coverage on lineage-defining genes.: The murine and humanized ChromHMM states were uploaded to the UCSC genome browser. Chondrogenic genes with the highest number of transitions (Supp.Fig. B13) are depicted (Tgfb1 (A), Shox2 (B), Osr1 (C) and Col27a1 (D)). Transitions from the ActGene (dark green) or ActProm (light green) to the RepActPro state (orange) were mainly detected in the promoter region. No significant differences were detected between the murine and the humanized model. [file 13072_2025_594_MOESM7_ESM.pdf]

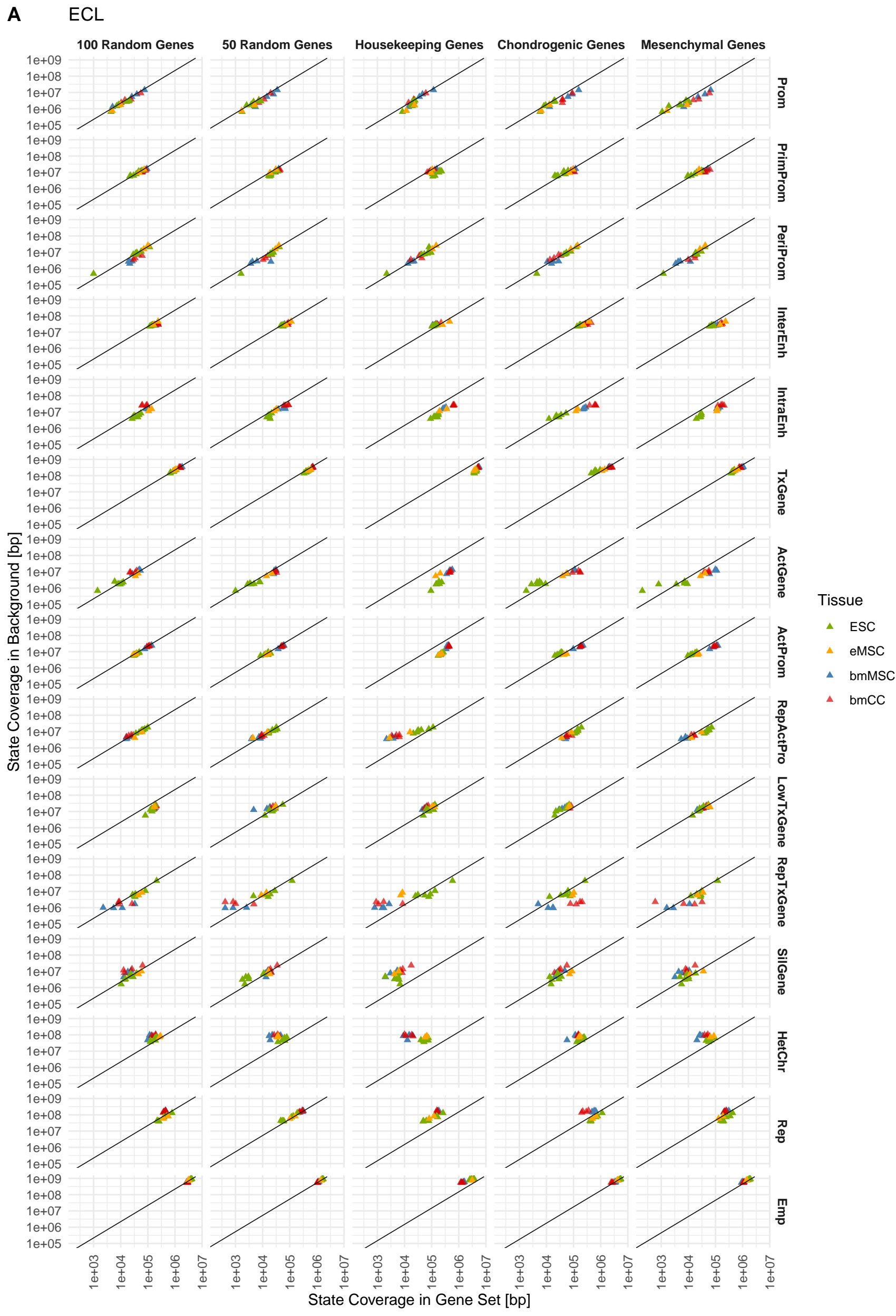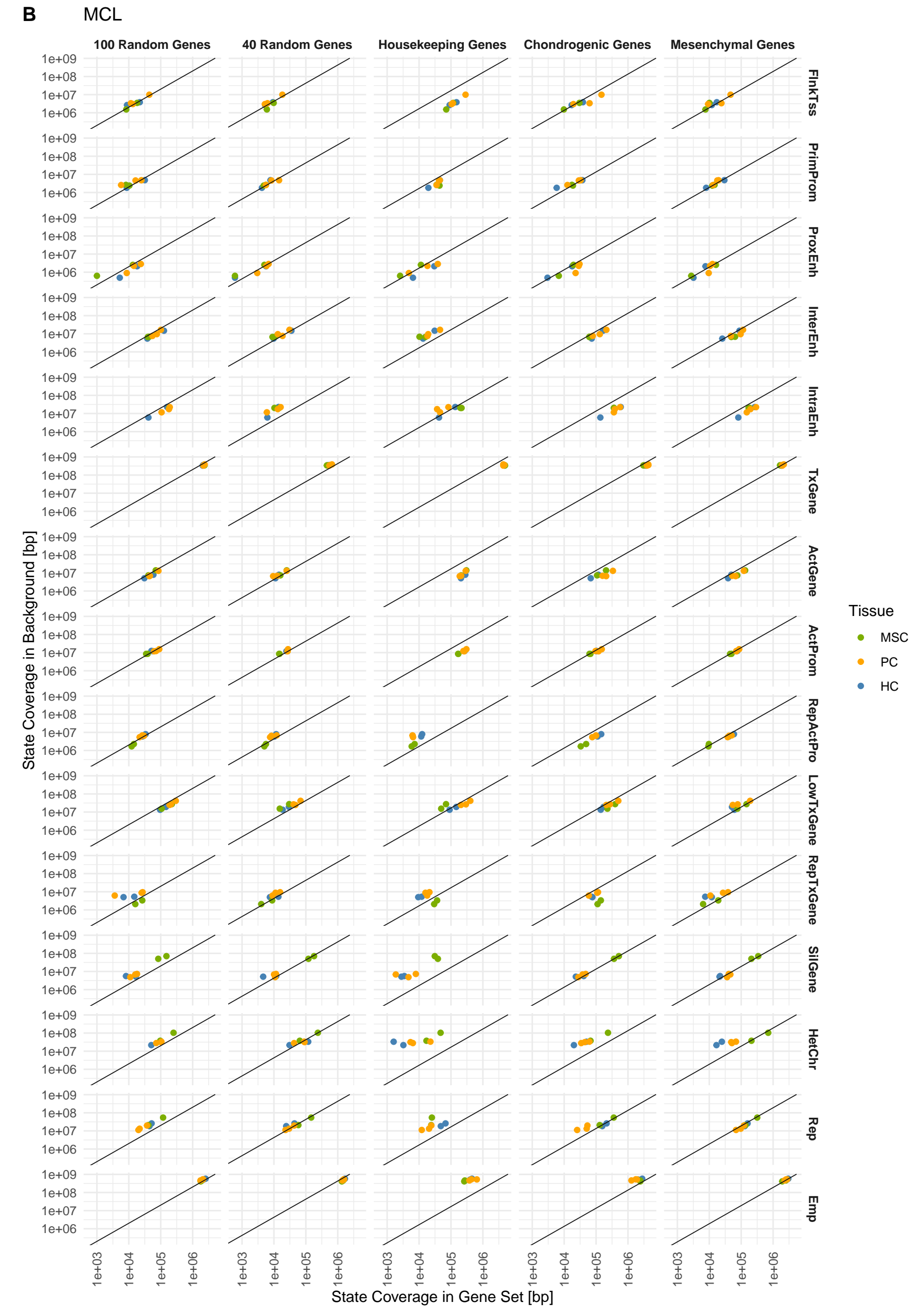

Supplement: Supplementary file 8 — Fig. B8 Comparison of the coverage of ChromHMM states on gene sets of interest to background genes for the ECL (A) and MCL (B).: Coverage in base pairs (bp) of a given ChromHMM state (row) on a given gene set (column) plotted against the coverage on all genes (background). The black line (slope = [length of all background genes] / [length of all genes within a set], intercept = 0) delineates an equal proportional coverage between the background and the gene set of interest. See also Fig. 2. [file 13072_2025_594_MOESM8_ESM.pdf]

**A****ECL**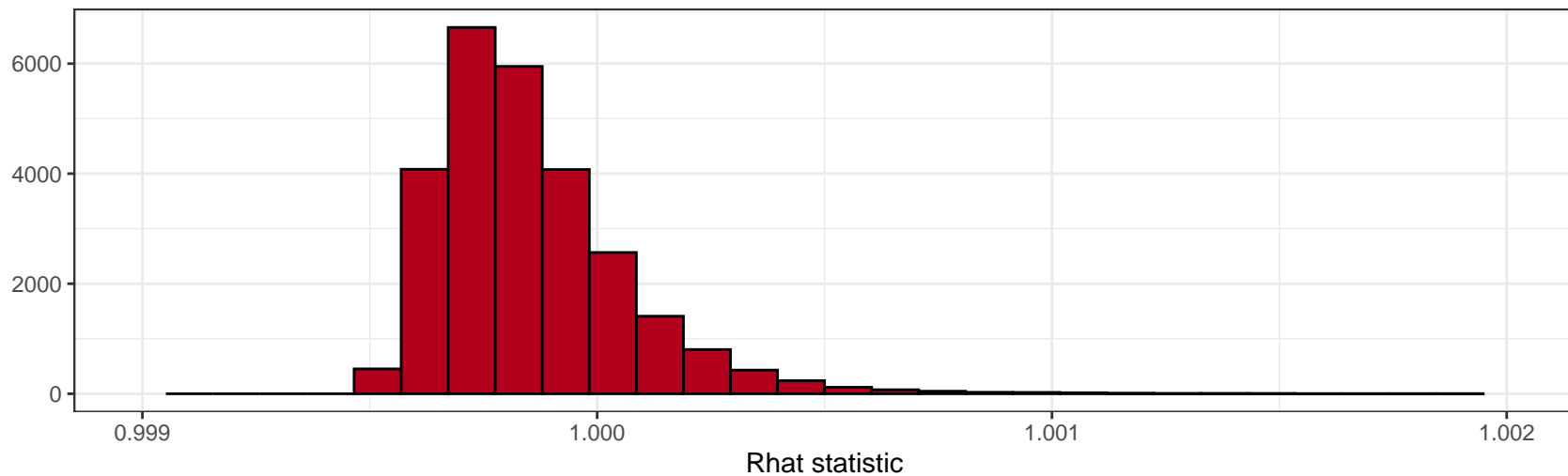**B****MCL**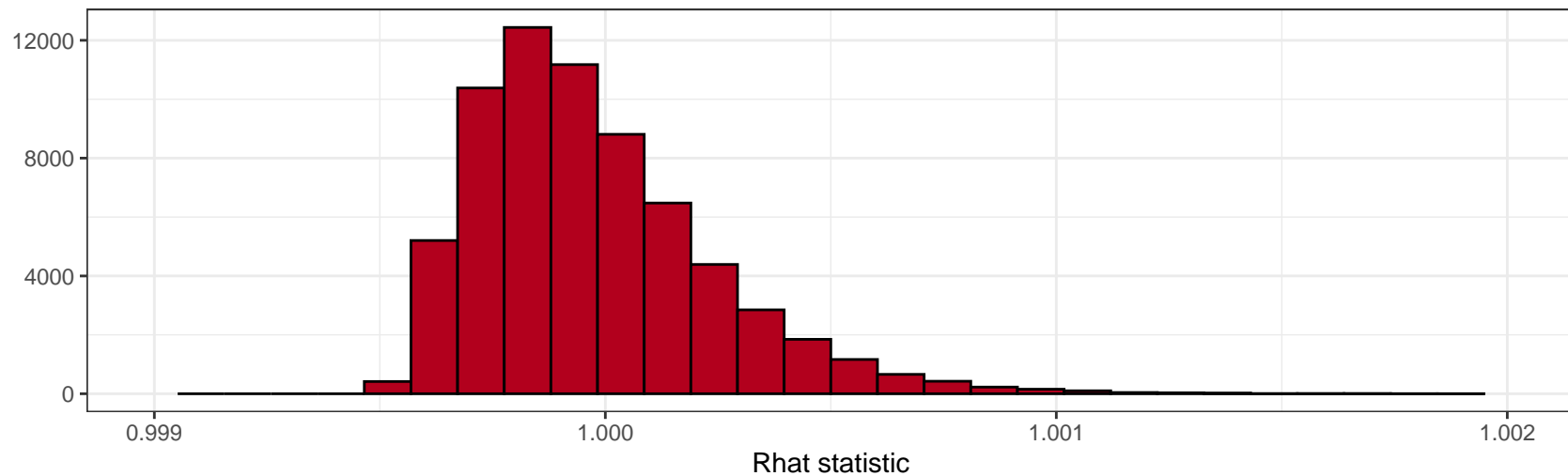

Supplement: Supplementary file 9 — Fig. B9 Convergence diagnostic for the Bayesian transition model.: Models for ECL and MCL have well-mixed chains with R-values close to 1, below the recommended upper bound of about 1.05 [61]. [file 13072_2025_594_MOESM9_ESM.pdf]

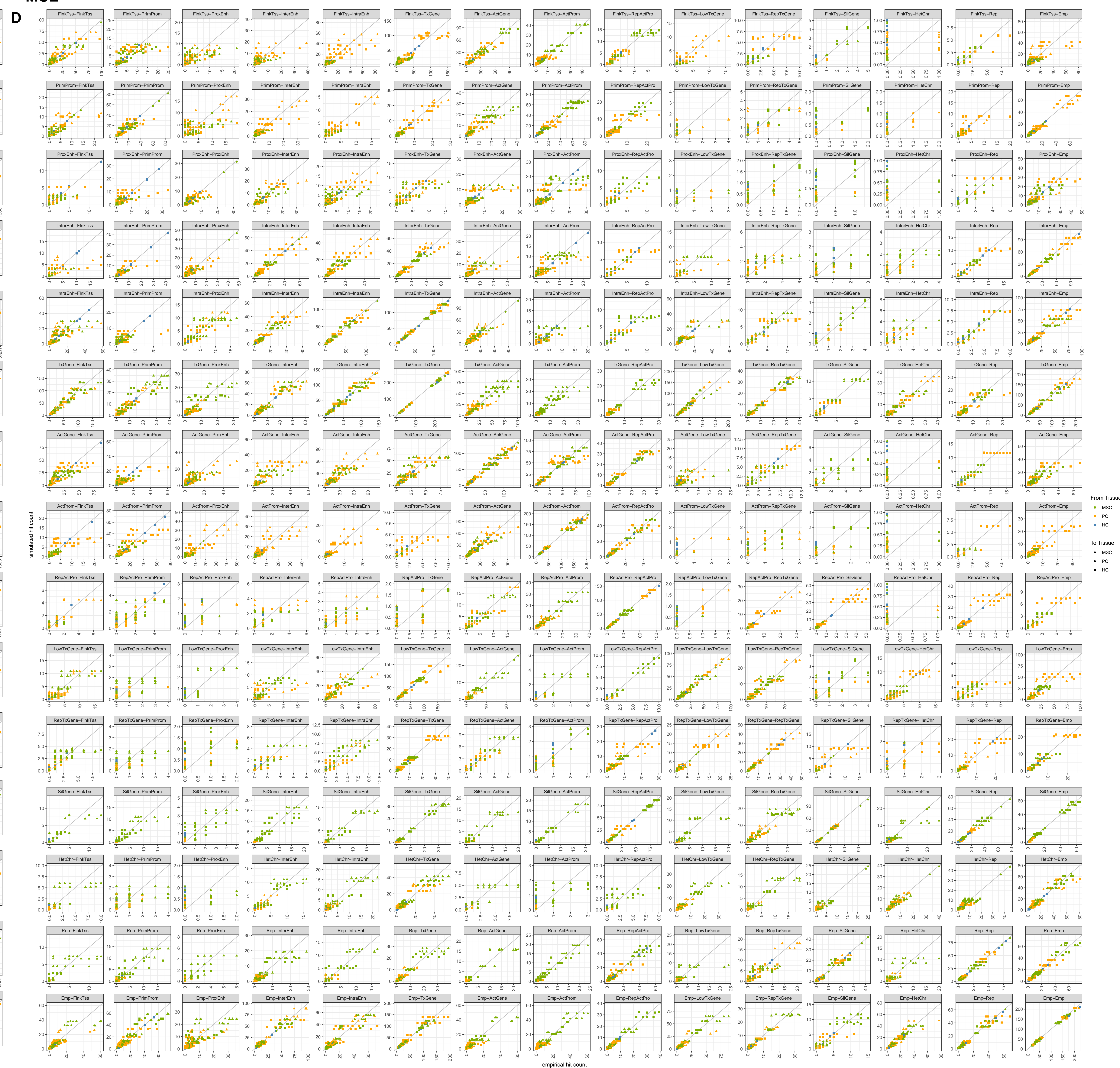

Supplement: Supplementary file 10 — Fig. B10 Posterior predictive checks for the Bayesian transition models for ECL (A-B) and MCL (C-D).: Contrasting the number of genes with a given transition (x-axes) against the average expected number of genes based on the model’s parameter estimates (y-axes). Each data point represents a specific tissue pair (indicated by color and shape) and gene set of interest. The variation along the x-axis reflects differences between replicates, grouped by a common y-value, which represents the model’s estimate of the average expected number of genes. Due to the vastly different scale, the data are split into measurements and estimates of the background (A, C) and measurements and estimates of the gene sets (B, D).The gray line indicates a perfect match between the measurements and the average estimates. For an overview of the model’s performance, see Supp. Fig. B11. [file 13072_2025_594_MOESM10_ESM.pdf]

**A**

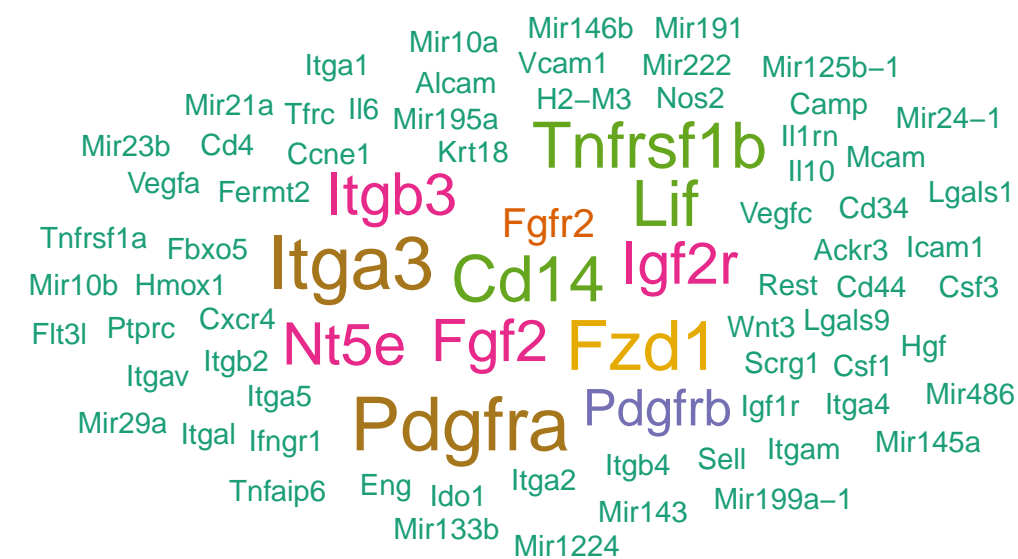

**C**

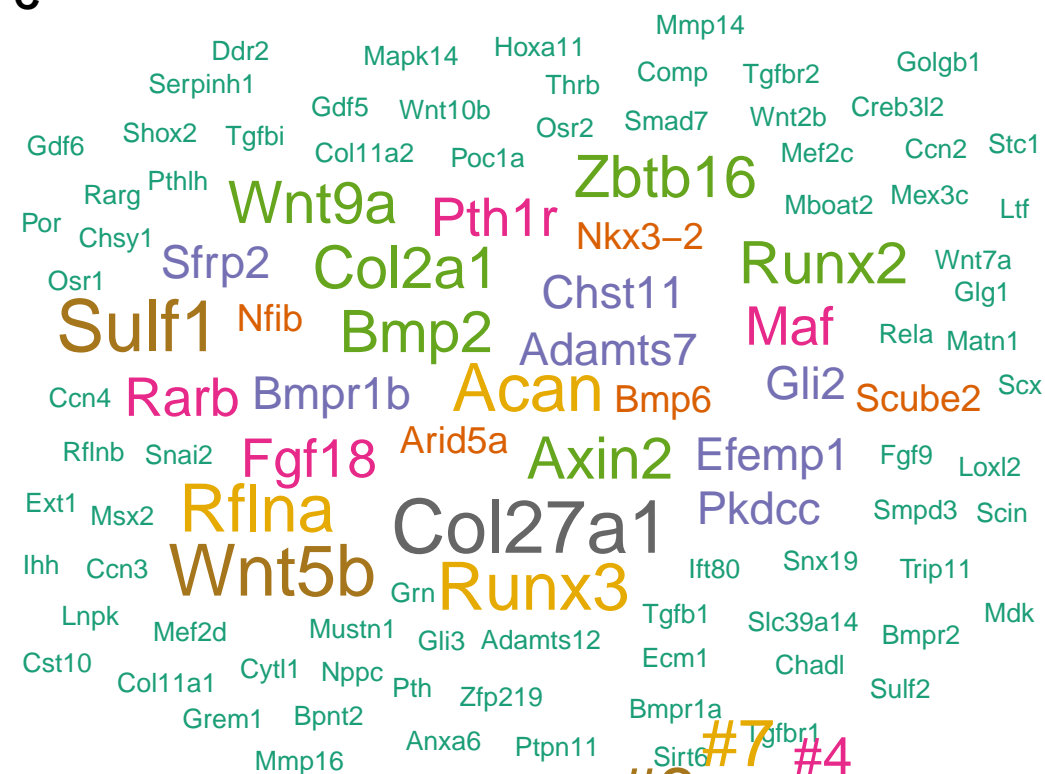

A word cloud of gene symbols including Bmpria, Sirt6, Tgfb1, #7, #4, #8, #2, #0, #3, #6, and #10.

## B

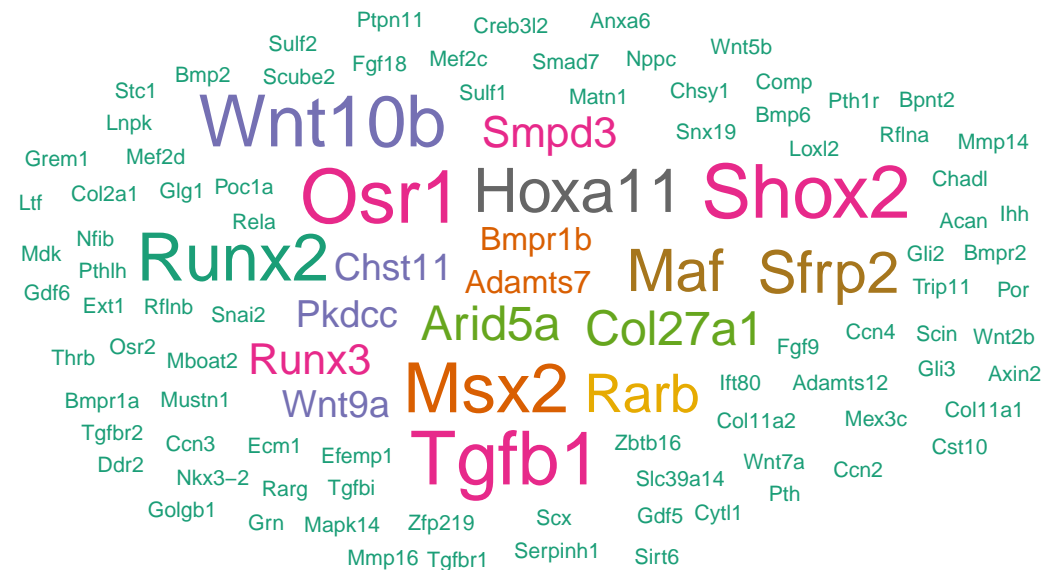

Supplement: Supplementary file 13 — Fig. B13 Occurrence rate for the gain of the repressive mark H3K27me3 on activating states in mesenchymal and chondrogenic genes of the MCL data.: In the word cloud, the size and color indicate in how many combinations of samples any given gene shows the transition from the ActProm or ActGene to the RepActPro state. Numbers show the combined occurrence of both transitions on each gene. (A) Transitions from MSC to PC on mesenchymal genes. With 2 and 3 replicates respectively, this leads to a maximum of 12 possible occurrences for a given gene (6 combinations of replicates for each of the 2 transitions). (B) Transitions from MSC to PC or HC on chondrogenic genes. With 2, 3, and 2 replicates respectively, this leads to a maximum of 24 possible occurrences. (C) Transition from PC to HC on chondrogenic genes. With 3 and 2 replicates respectively, this leads to a maximum of 12 possible occurrences. See Supp. Tab. A3 for the data visualised in this figure. [file 13072_2025_594_MOESM13_ESM.pdf]
