# Supplementary material for: Bath: a Bayesian approach to analyze epigenetic transitions reveals a dual role of H3K27me3 in chondrogenesis
Source: Epigenetics Chromatin. 2025 Jun 27;18:38. doi: 10.1186/s13072-025-00594-6 (PMC12203727; doi:10.1186/s13072-025-00594-6)
Supplement: Supplementary file 11 — Fig. B11 Summary of Posterior predictive checks for the Bayesian transition models. The mean difference between the estimated and observed number of genes (y-axes) is plotted against the observed gene count (x-axes), for ECL (A) and MCL (B). Each datapoint represents a specific combination of gene set, tissue pair, and state transition. The majority of combinations show a difference very close to 0. Very small and large gene counts exhibit systematic over- or underrepresentation compared to the observed data, respectively. This is expected, as the model is designed to be more conservative with extreme values. When the observed data are less reliable (e.g. fewer replicates, as seen for MSC to MSC and HC to HC, with only two replicates each), the model becomes more cautious with extreme values, resulting in an increased difference. For a more detailed illustration of the PPC, see Supp. Fig. B10. [file 13072_2025_594_MOESM11_ESM.pdf]

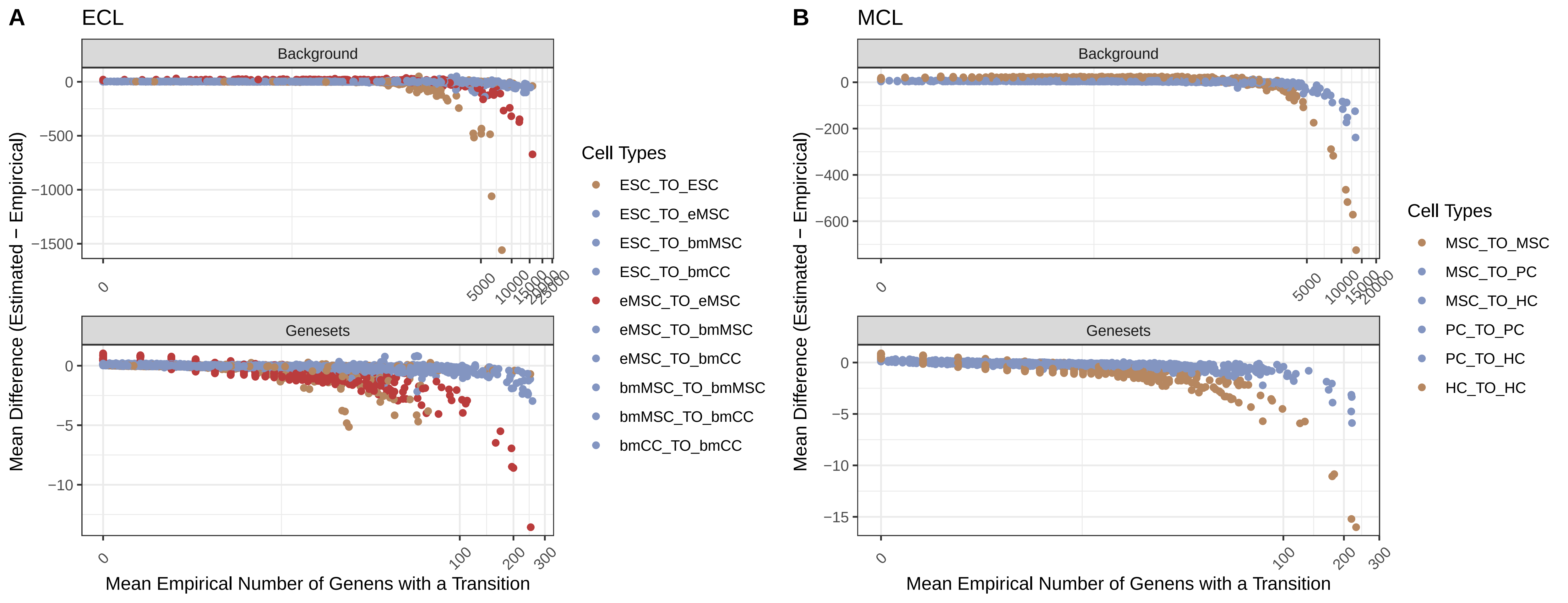

A
ECL
Background
Genesets
Mean Difference (Estimated − Empirical)
Mean Empirical Number of Genens with a Transition
0
−500
−1000
−1500
5000
10000
15000
20000
25000
0
−5
−10
100
200
300
Cell Types
ESC_TO_ESC
ESC_TO_eMSC
ESC_TO_bmMSC
ESC_TO_bmCC
eMSC_TO_eMSC
eMSC_TO_bmMSC
eMSC_TO_bmCC
bmMSC_TO_bmMSC
bmMSC_TO_bmCC
bmCC_TO_bmCC
B
MCL
Background
Genesets
Mean Difference (Estimated − Empirical)
Mean Empirical Number of Genens with a Transition
0
−200
−400
−600
5000
10000
15000
20000
0
−5
−10
−15
100
200
300
Cell Types
MSC_TO_MSC
MSC_TO_PC
MSC_TO_HC
PC_TO_PC
PC_TO_HC
HC_TO_HC
