# Supplementary material for: Bath: a Bayesian approach to analyze epigenetic transitions reveals a dual role of H3K27me3 in chondrogenesis
Source: Epigenetics Chromatin. 2025 Jun 27;18:38. doi: 10.1186/s13072-025-00594-6 (PMC12203727; doi:10.1186/s13072-025-00594-6)
Supplement: Supplementary file 12 — Fig. B12 CLES values calculated for each transitional enrichment and depletion (CLES) for every state and cell type combination for each set of genes and type of dataset (ECL or MCL).: The rows indicate the originating and the columns the target states of the respective cell types. Colored axes classify the states as empty (gray), repressive (red), repressed-active (orange), and activating (green). The blue and red squares demarcate the transitions highlighted in Fig. 3 and Fig. 4, respectively. [file 13072_2025_594_MOESM12_ESM.pdf]

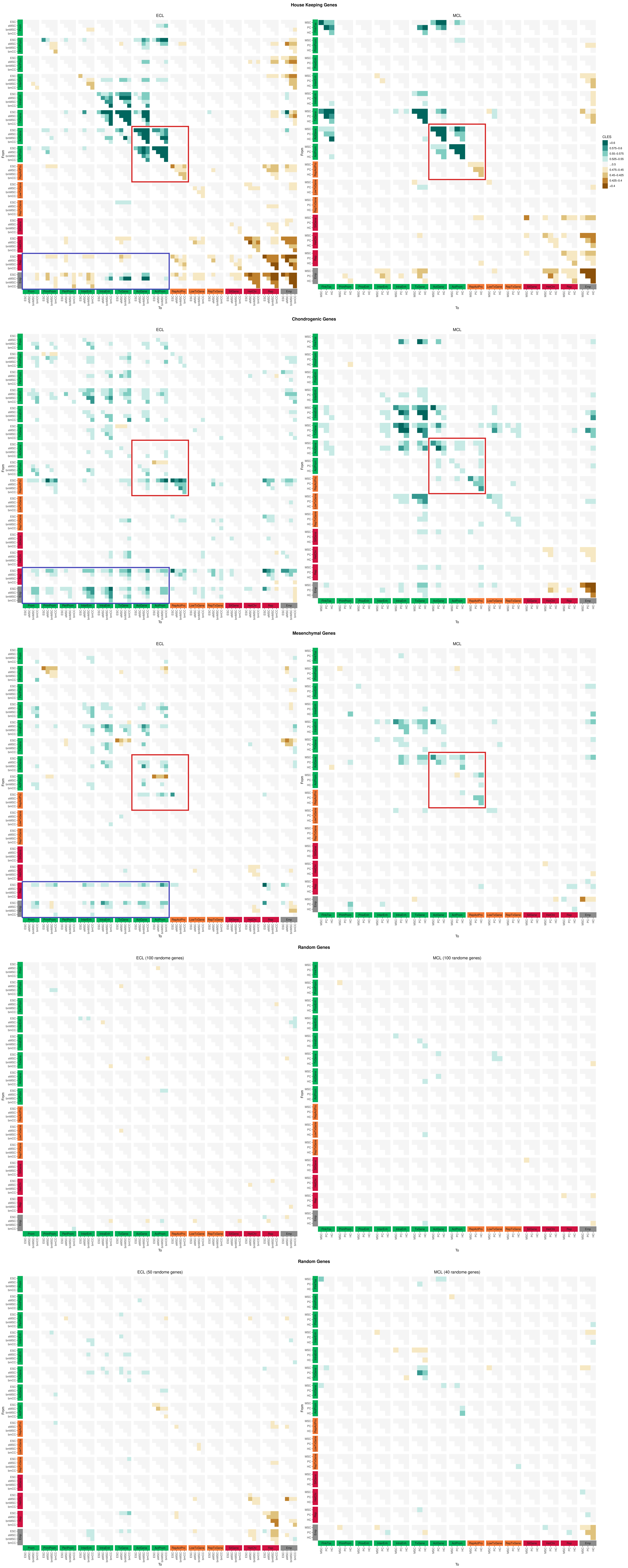
House Keeping Genes
ECL
MCL
Chondrogenic Genes
ECL
MCL
Mesenchymal Genes
ECL
MCL
Random Genes
ECL (100 randome genes)
MCL (100 randome genes)
Random Genes
ECL (50 randome genes)
MCL (40 randome genes)
From
To
ESC
eMSC
bmMSC
bmCC
MSC
PC
HC
FlnkTss
PrimProm
ProxEnh
InterEnh
IntraEnh
TxGene
ActGene
ActProm
RepActPro
LowTxGene
RepTxGene
SilGene
HetChr
Rep
Emp
CLES
>0.6
0.575–0.6
0.55–0.575
0.525–0.55
...0.5
0.475–0.45
0.45–0.425
0.425–0.4
<0.4
